# Supplementary material for: Antimicrobial resistance in topical treatments for microbial keratitis: protocol for a systematic review and meta-analysis
Source: BMJ Open. 2023 Mar 7;13(3):e069338. doi: 10.1136/bmjopen-2022-069338 (PMC10008341; doi:10.1136/bmjopen-2022-069338)
Supplement: Supplementary data [file bmjopen-2022-069338supp001.pdf]

Supplementary figure 1
Search strategy

MEDLINE
1. keratitis/ or acanthamoeba keratitis/
2. keratitis.tw.
3. (fung$ adj3 infect$ adj3 eye$).tw.
4. (fung$ adj3 infect$ adj3 ocular).tw.
5. (bacteria$ adj3 infect$ adj3 eye$).tw.
6. (bacteria$ adj3 infect$ adj3 ocular).tw.
7. or/1-6
8. exp Drug Resistance, Microbial/
9. ((antimicrobial or antifungal or increase$) adj2 resistan$).tw.
10. "AMR".tw.
11. Microbial Sensitivity Tests/
12. (drug adj2 (resistan$ or suscept$)).tw.
13. (minimum adj1 inhibit$ adj1 concentration$).tw.
14. "MIC".tw.
15. breakpoint.tw.
16. or/8-15
17. prevalence/
18. prevalence.tw.
19. case-control studies/ or retrospective studies/ or cohort studies/ or follow-up studies/ or longitudinal studies/ or prospective studies/ or cross-sectional studies/ or observational study/
20. (case adj1 control$ adj3 (stud$ or trial$)).tw.
21. (cross adj1 sectional$ adj3 (stud$ or trial$)).tw.
22. (follow adj1 up adj3 (stud$ or trial$)).tw.
23. ((prospective or retrospective or cohort or longitudinal or observational) adj3 (stud$ or trial$)).tw.
24. clinical trial/ or control groups/ or double-blind method/ or meta-analysis as topic/ or network meta-analysis/ or random allocation/ or single-blind method/
25. randomized controlled trial/ or controlled clinical trials as topic/ or randomized controlled trials as topic/ or comparative study/ or evaluation studies/ or meta- analysis/ or "systematic review"/
26. random$.tw.
27. or/17-26
28. 7 and 16 and 27
29 (animal or animals or mouse or mice or rat or rats or rabbit$ or dog or dogs or canine or cat or cats or pig or pigs or horse or horses or veterinary).tw.
30 28 not 29

Embase
1. keratitis/ or bacterial keratitis/ or virus keratitis/ or Acanthamoeba keratitis/ or microbial keratitis/ or suppurative keratitis/ or amebic keratitis/
2. keratitis.tw.
3. (fung$ adj3 infect$ adj3 eye$).tw.
4. (fung$ adj3 infect$ adj3 ocular).tw.
5. (bacteria$ adj3 infect$ adj3 eye$).tw.
6. (bacteria$ adj3 infect$ adj3 ocular).tw.
7. or/1-6
8. antibiotic resistance/ or antibiotic sensitivity/ or drug resistance/

9. aminoglycoside resistance/ or beta-lactam resistance/ or chloramphenicol resistance/ or fluoroquinolone resistance/
10. ((antimicrobial or antifungal or increase$) adj2 resistan$).tw.
11. "AMR".tw.
12. microbial sensitivity test/
13. (drug adj2 (resistan$ or suscept$)).tw.
14. minimum inhibitory concentration/
15. (minimum adj1 inhibit$ adj1 concentration$).tw.
16. "MIC".tw.
17. breakpoint.tw.
18. or/8-17
19. prevalence/
20. prevalence.tw.
21. controlled clinical trial/
22. case control study/
23. cohort analysis/
24. follow up/
25. longitudinal study/
26. observational study/
27. prospective study/
28. retrospective study/
29. cross-sectional study/
30. control group/
31. "meta analysis (topic)"/
32. network meta-analysis/
33. randomization/
34. single blind procedure/
35. double blind procedure/
36. control group/
37. controlled clinical trial/
38. clinical study/
39. clinical trial/
40. "clinical trial (topic)"/
41. "controlled clinical trial (topic)"/
42. "randomized controlled trial (topic)"/
43. comparative study/
44. evaluation study/
45. randomized controlled trial/
46. meta analysis/
47. "systematic review"/
48. "systematic review (topic)"/
49. (case adj1 control$ adj3 (stud$ or trial$)).tw.
50. (cross adj1 sectional$ adj3 (stud$ or trial$)).tw.
51. (follow adj1 up adj3 (stud$ or trial$)).tw.
52. ((prospective or retrospective or cohort or longitudinal or observational) adj3 (stud$ or trial$)).tw.
53. random$.tw.
54. or/19-53
55. 7 and 18 and 54
56. (animal or animals or mouse or mice or rat or rats or rabbit$ or dog or dogs or canine or cat or cats or pig or pigs or horse or horses or veterinary).tw.

57. 55 not 56

Cochrane Library

#1 MeSH descriptor: [Keratitis] this term only
#2 MeSH descriptor: [Acanthamoeba Keratitis] this term only
#3 keratitis
#4 fung* NEAR/3 infect* NEAR/3 eye
#5 fung* NEAR/3 infect* NEAR/3 ocular
#6 bacteria* NEAR/3 infect* NEAR/3 eye*
#7 bacteria* NEAR/3 infect* NEAR/3 ocular
#8 #1 or #2 or #3 or #4 or #5 or #6 or #7
#9 MeSH descriptor: [Drug Resistance, Microbial] explode all trees
#10 (antimicrobial or antifungal or increase*) NEAR/2 resistan*
#11 AMR
#12 MeSH descriptor: [Microbial Sensitivity Tests] this term only
#13 drug NEAR/2 (resistan* or suscept*)
#14 minimum NEXT inhibit* NEXT concentration*
#15 MIC
#16 breakpoint
#17 #9 or #10 or #11 or #12 or #13 or #14 or #15 or #16
#18 #8 and #17

Web of Science

#19 not #20
TS= (animal OR animals OR mouse OR mice OR rabbit* OR dog OR dogs OR canine OR horse OR horses OR veterinary)
#6 AND #11 AND #18
#17 OR #16 OR #15 OR #14 OR #13 OR #12
TS= (clinical OR comparative NEAR/3 (stud* OR trial*))
TS= (random* OR meta NEAR/1 analysis OR systematic NEAR/1 review)
TS= (follow NEAR/2 up NEAR/3 (stud* OR trial*))
TS= (cross NEAR/2 sectional* NEAR/3 (stud* OR trial*))
TS= (case NEAR/2 control* NEAR/3 (stud* OR trial*))
TS= (prevalence OR retrospective OR prospective OR cohort OR observational OR longitudinal)
#7 OR #8 OR #9 OR #10
TS= (drug NEAR/2 (resistan* or suscept*))
TS= ((antimicrobial OR antifungal OR increase*) NEAR/2 resistan*)
TS=(Microbial Sensitivity Tests)
TS=(Microbial Drug Resistance)
#1 OR #2 OR #3 OR #4 OR #5
TS= keratitis
TS= (fung* NEAR/3 infect* NEAR/3 eye*)
TS= (fung* NEAR/3 infect* NEAR/3 ocular)
TS= (bacteria* NEAR/3 infect* NEAR/3 eye*)
TS= (bacteria* NEAR/3 infect* NEAR/3 ocular)

Clinicaltrials.gov

(antimicrobial OR antifungal OR antibiotic OR drug) AND resistance | Keratitis

WHO ICTRP

Keratitis AND antimicrobial AND resistance OR Keratitis AND antifungal AND resistance OR Keratitis AND antibiotic AND resistance OR Keratitis AND drug AND resistance
