# Supplementary material for: Antimicrobial resistance in topical treatments for microbial keratitis: protocol for a systematic review and meta-analysis
Source: BMJ Open. 2023 Mar 7;13(3):e069338. doi: 10.1136/bmjopen-2022-069338 (PMC10008341; doi:10.1136/bmjopen-2022-069338)
Supplement: Supplementary data [file bmjopen-2022-069338supp002.pdf]

Supplementary Figure 2: Risk of Bias Assessment

	**Risk of bias item**	**Criteria for answers**
1	Was the study's target population a close representation of the national population in relation to relevant variables?	Yes (LOW RISK)<br>The study's target population was a close representation of the national or regional population *e.g., population-based study or sampling of cases of microbial keratitis from representative settings.*<br>No (HIGH RISK)<br>The study's target population was clearly not representative of the national population. *e.g., sampling of cases attending tertiary referral center or cases selected from a population that had failed to improve with prior therapy with antimicrobials*, *selection of larger ulcers, exclusions of small peripheral ulcers or of impending or actual corneal perforation.*
2	Was the sampling frame a true or close representation of the target population?	Yes (LOW RISK): The sampling frame was a true or close representation of the target population, *e.g., an acceptable and standardized definition of microbial keratitis was used, including definitions of pathogens, standardized criteria for definition and selection of isolate type, and definition of significant growth.*<br><br>No (HIGH RISK): The sampling frame was NOT a true or close representation of the target population, e.g., *potential 'non-pathogens' were excluded. Unclear the basis for the clinical diagnosis. Definitions of pathogens and isolates are not standardized.*
3	Was some form of random selection used to select the sample, OR was a census undertaken?	Yes (LOW RISK): A census was undertaken, OR, some form of random selection was used to select the sample (e.g. simple random sampling, stratified random sampling, cluster sampling, systematic sampling), e.g. *random sample of a consecutive series of patients, or inclusion of a consecutive series of patients*<br><br>No (HIGH RISK): A census was NOT undertaken, AND some form of random selection was NOT used to select the sample *eg unclear how cases selected or unclear if consecutive or not*
4	Was the likelihood of non-response bias minimal?	Yes (LOW RISK): The response rate for the study was ≥75%, OR, an analysis was performed that showed no

		significant difference in relevant demographic characteristics between responders and nonresponders<br><br>No (HIGH RISK): The response rate was <75%, and if any analysis comparing responders and non-responders was done, it showed a significant difference in relevant demographic characteristics between responders and non-responders.
5	Were data collected directly from the subjects (as opposed to a proxy)?	Yes (LOW RISK): All data were collected directly from the subjects. *It is likely that data will be collected directly from all participants so this item may not be relevant and will be marked low risk of bias for all studies.*<br><br>No (HIGH RISK): In some instances, data were collected from a proxy. *Difficult to envisage a study where this would be the case.*
6	Was an acceptable and standardized case definition for AMR used in the study?	Yes (LOW RISK): An acceptable case definition was used, e.g., *definition of AMR clearly described and standardized (between geographical locations and over time if applicable)*<br><br>No (HIGH RISK): An acceptable case definition was NOT used. *e.g., unclear definition of AMR and/or definition not consistently applied in different groups of patients or over time.*
7	Was the study instrument that measured the parameter of interest shown to have validity and reliability?	Yes (LOW RISK): The study instrument had been shown to have reliability and validity (if this was necessary), e.g., test-retest, piloting, validation in a previous study, etc., *e.g., method of measuring AMR was valid and reliable i.e., valid laboratory systems for the identification of isolates and measurement of AMR or MIC.*<br><br>No (HIGH RISK): The study instrument had NOT been shown to have reliability or validity (if this was necessary), *e.g., method of measuring AMR was not clearly described or not validated.*
8	Was the same mode of data collection used for all subjects?	Yes (LOW RISK): The same mode of data collection was used for all subjects, e.g., *collection methodologies for the identified cases and measurement of resistance or MIC were adequately described and consistent during the course of the study.*

		No (HIGH RISK): The same mode of data collection was NOT used for all subjects, e.g., *collection methodologies for the identified and measurement of resistance or MIC not clearly described and/or inconsistent during the course of the study.*
9	Was the length of the shortest prevalence period for the parameter of interest appropriate?	Yes (LOW RISK): The shortest prevalence period for the parameter of interest was appropriate (e.g., point prevalence, one-week prevalence, one-year prevalence), e.*g., the study was done over an appropriate time period*<br><br>No (HIGH RISK): The shortest prevalence period for the parameter of interest was not appropriate (e.g., lifetime prevalence), e.g., *there may be seasonal variation in the proportions of isolates if the sampling interval was less than 12 months.*
10	Were the numerator(s) and denominator(s) for the parameter of interest appropriate?	Yes (LOW RISK): The paper presented appropriate numerator(s) AND denominator(s) for the parameter of interest, e.g., *it is clear that numerators and denominators are correct including grouping of species and documentation of potential pathogens/contaminants consistent in both groups.*<br><br>No (HIGH RISK): The paper did present numerator(s) AND denominator(s) for the parameter of interest but one or more of these were inappropriate
	Summary risk of bias	LOW RISK OF BIAS: Further research is very unlikely to change our confidence in the estimate.<br><br>MODERATE RISK OF BIAS: Further research is likely to have an important impact on our confidence in the estimate and may change the estimate.<br><br>HIGH RISK OF BIAS: Further research is very likely to have an important impact on our confidence in the estimate and is likely to change the estimate.

Adapted from Hoy et al.(Hoy et al. 2012)

Hoy, Damian, Peter Brooks, Anthony Woolf, Fiona Blyth, Lyn March, Chris Bain, Peter Baker, Emma Smith, and Rachelle Buchbinder. 2012. “Assessing Risk of Bias in Prevalence Studies: Modification of an Existing Tool and Evidence of Interrater Agreement.” *Journal of Clinical Epidemiology* 65 (9): 934–39.
