# Supplementary material for: Antimicrobial resistance in topical treatments for microbial keratitis: protocol for a systematic review and meta-analysis
Source: BMJ Open. 2023 Mar 7;13(3):e069338. doi: 10.1136/bmjopen-2022-069338 (PMC10008341; doi:10.1136/bmjopen-2022-069338)
Supplement: Author's manuscript [file bmjopen-2022-069338.draft_revisions.pdf]

BMJ Open

BMJ Open is committed to open peer review. As part of this commitment we make the peer review history of every article we publish publicly available.

When an article is published we post the peer reviewers' comments and the authors' responses online. We also post the versions of the paper that were used during peer review. These are the versions that the peer review comments apply to.

The versions of the paper that follow are the versions that were submitted during the peer review process. They are not the versions of record or the final published versions. They should not be cited or distributed as the published version of this manuscript.

BMJ Open is an open access journal and the full, final, typeset and author-corrected version of record of the manuscript is available on our site with no access controls, subscription charges or pay-per-view fees (http://bmjopen.bmj.com).

If you have any questions on BMJ Open's open peer review process please email info.bmjopen@bmj.com

BMJ Open

Antimicrobial resistance in topical treatments for microbial keratitis: protocol for a systematic review

Journal:	*BMJ Open*
Manuscript ID	bmjopen-2022-069338
Article Type:	Protocol
Date Submitted by the Author:	20-Oct-2022

Complete List of Authors:	Tuft, Stephen; Moorfields Eye Hospital NHS Foundation Trust; Joint Library of Ophthalmology Moorfields Eye Hospital and UCL Institute of Ophthalmology<br>Evans, Jennifer; LSHTM, Faculty of Infectious and Tropical Diseases<br>Gordon, Iris; LSHTM, Department of Infectious and Tropical Diseases<br>Leck, Astrid; LSHTM, Department of Infectious and Tropical Diseases<br>Stone, Neil; University College London Hospitals NHS Foundation Trust, Department of Clinical Microbiology<br>Neal, Timothy; Royal Liverpool University Hospital<br>Macleod, David; LSHTM, Department of Medical Statistics<br>Kaye, Stephen; University of Liverpool<br>Burton, Matthew J; LSHTM, Faculty of Infectious and Tropical Diseases

Keywords:	MICROBIOLOGY, Corneal and external diseases < OPHTHALMOLOGY, EPIDEMIOLOGY, Public health < INFECTIOUS DISEASES

SCHOLARONE™
Manuscripts

Antimicrobial resistance in topical treatments for microbial keratitis: protocol for a systematic review

Stephen Tuft[1,2], Jennifer R Evans[3,4], Iris Gordon[3], Astrid Leck[3], Neil Stone[5], Timothy Neal[6], David Macleod[3,7], Stephen Kaye[8], Matthew J Burton[1,3].

[1]Moorfields Eye Hospital, 162 City Road, London EC1V 2PD

[2]UCL Institute of Ophthalmology, 11-43 Bath Street, London EC1V 9EL

[3]International Centre for Eye Health, London School of Hygiene & Tropical Medicine, Keppel St, London WC1E 7HT

[4]Centre for Public Health, Queen's University Belfast, Institute of Clinical Science, Block A, Royal Victoria Hospital, Belfast BT12 6BA

[5]Department of Clinical Microbiology, University College London Hospitals NHS Foundation Trust, 250 Euston Road, London NW1 2PG.

[6]Department of Clinical Microbiology, Liverpool Clinical Laboratories, Liverpool University Hospital NHS Foundation Trust, Prescot Street, Liverpool L7 8XP.

[7]MRC International Statistics and Epidemiology Group, London School of Hygiene & Tropical Medicine, Keppel St, London WC1E 7HT

[8]Department of Eye and Vision Sciences, University of Liverpool, 6 West Derby Street, Liverpool L7 8TX.

Corresponding author: Professor Stephen Tuft

0044 0207 253 2341

Email: s.tuft@ucl.ac.uk

Word count: 3696

Key words: Cornea, keratitis, fungus, acanthamoeba, microbial resistance, systematic review

For peer review only

ABSTRACT

Introduction: There is evidence for increased resistance against the antimicrobials used to treat keratitis. This review aims to provide global and regional prevalence estimates of antimicrobial resistance in corneal isolates, and the range of minimum inhibitory concentrations (MIC) with their associated resistance breakpoints.

Methods and analysis: We report this protocol following PRISMA-P guidelines. We will conduct an electronic bibliographic search in MEDLINE, EMBASE, Web of Science, and the Cochrane Library. Eligible studies will report in any language data for the resistance or MIC for antimicrobials against organisms isolated from suspected microbial keratitis. There will be no time restrictions on the date of publication. Screening for eligible studies, assessment of risk of bias and data extraction will be conducted by two reviewers independently, using predefined inclusion criteria and pre-piloted data extraction forms. We will resolve disagreements between the reviewers by discussion and, if required, a third (senior) reviewer will arbitrate. We will assess the risk of bias using a tool validated in prevalence studies. The certainty of the evidence will be assessed using the Grades of Recommendation, Assessment, Development and Evaluation (GRADE) approach. Pooled proportion estimates will be calculated using a random-effects model. Heterogeneity will be assessed using the I^2 statistic. We will explore regional differences and temporal trends.

Ethics approval and dissemination: Ethics approval is not required as this is a protocol for a systematic review of published data. The findings of this review will be published in an open-access, peer-reviewed journal.

Prospero registration number: to be confirmed

- **What is already known on this topic** - Antimicrobial resistance (AMR) is a global phenomenon that has made some systemic infections difficult to treat. An increase in the

prevalence of resistance to fluoroquinolones, beta-lactams and aminoglycosides has also been reported in common ophthalmic isolates.

- **What this study adds** - We plan to document systematically the global prevalence of AMR with associated minimum inhibitory concentrations for isolates from cases of microbial keratitis.
- **How this study might affect research, practice, or policy** - This study will provide benchmark data for clinicians, clinical scientists, and stakeholders. It is anticipated to highlight important microbial resistant patterns and trends, which could inform antimicrobial prescribing. It is also hoped that it will highlight significant data gaps and encourage a fuller, more standardised reporting approach.

INTRODUCTION

Microbial keratitis (MK) is a common corneal infection treated with topical antimicrobials. The isolated organisms include bacteria, fungi, protozoa, and viruses, either in isolation or in combination. The relative proportions of pathogens (viruses, bacteria, parasites, fungi) vary widely between geographic regions, with fungal keratitis particularly prevalent in low- and middle-income countries and equatorial areas.[1] Published figures for the incidence of MK include 113 per 100,000 person-years in South India[1–3] and 799 per 100,000 person-years from Nepal.[4] A population-based study in China estimated the prevalence of past or active microbial keratitis to be 192 (95% CI 171–213) per 100,000, with a prevalence of presumed viral keratitis of 110, bacterial keratitis 75, and fungal keratitis 7 per 100,000.[2] The incidence figures for MK reported from high-income countries since 1995 are much lower with estimates of 4.5-37.7 cases per 100,000 person-years in the US, UK, Australia and Taiwan,[3] which follows a marked increase in cases from the 1970s associated with the widespread introduction of contact lens wear.[4]

For peer review only

The relative frequency and distribution of the potential bacterial pathogens isolated from corneal ulcers are usually derived from retrospective data rather than prospective surveys. The proportions vary widely between reports and according to the definition used for a significant isolate. In a recent meta-analysis of 38 studies, the most common isolates were *Staphylococcus* spp. (including *Staphylococcus aureus* and coagulase-negative staphylococci) (41.4%, 95% confidence limits 36.2–46.7%), *Pseudomonas* spp. (17.0%, 13.9–20.7%), *Streptococcus* spp. (13.1%, 10.9–15.7%), *Corynebacterium* spp. (6.6%, 5.3–8.3%) and *Moraxella* spp. *(4.1%, 3.1–5.4%).[5] Streptococcus pneumoniae* and *Nocardia* spp. are more frequently reported in series from South India, where naso-lacrimal duct obstruction and agricultural trauma are major risk factors.[6–8] Differences in environmental temperature, humidity, occupation, associated viral disease and malnutrition affect the local prevalence pattern.[9] Ideally, all ulcers should be sampled, as defining a threshold ulcer size to justify corneal culture will introduce case selection bias that could affect the spectrum of isolates.[10,11] Agricultural trauma is the major risk for infection due to filamentous fungi and *Acanthamoeba* spp. in low-income countries, as opposed to contact lens wear in high-income regions.[12]

Antimicrobial resistance (AMR) in bacteria is an intrinsic or acquired characteristic encoded by genes that can be transferred between bacteria.[13] AMR is a global phenomenon that has made some systemic bacterial infections challenging to treat.[14–17] An increase in the prevalence of resistance to fluoroquinolones, beta-lactams and aminoglycosides has been reported in ophthalmic isolates.[6,16,18–22] Although *P. aeruginosa* susceptibility to either ciprofloxacin or moxifloxacin for corneal isolates is still approximately 80% worldwide,[6,21–24] several studies have highlighted the presence of high proportions of AMR bacteria in ocular infections, particularly in the US,[25] China,[26] and India.[27] A particularly sharp increase in resistance of *P. aeruginosa* to moxifloxacin occurred between 2007-9 in southern India, from 19% (95% CI 5.4% to 41.9%) to 52% (95% CI 29.8% to 74.3%) (P=0.024).[28] Two separate

For peer review only - http://bmjopen.bmj.com/site/about/guidelines.xhtml

20-year reviews from the US found an increase in methicillin-resistant *S. aureus* keratitis from 1993 to 2015.[21,29] Fluoroquinolone-resistance, methicillin-resistance and multidrug-resistant (MDR) bacteria, defined as acquired resistance to at least one agent in 3 or more antimicrobial classes, has also been reported in isolates from bacterial keratitis.[23,30–33] Some common ophthalmic pathogens, particularly *P. aeruginosa*, can show MDR,[34,35] with MDR *P. aeruginosa* emerging as a cause of bacterial keratitis in South Asia.[36,37] In South China, an increase in AMR in Gram-positive cocci was reported between 2010 to 2018, while the susceptibility of Gram-negative bacilli to fluoroquinolones and aminoglycosides was stable.[26]

Evidence for a change in AMR to the agents used to treat fungal or acanthamoeba keratitis is less certain. Susceptibility testing for antifungal agents is usually only performed in large hospitals or national reference laboratories,[38] and susceptibility testing for antiamoebic agents is rarely performed. The prevalence of acquired resistance to antifungal agents are generally lower than antibiotic resistance, but the treatment options are more limited.[39,40] Most data on the prevalence of resistance to antifungal agents is derived from testing isolates from systemic infections, with relatively few studies of corneal isolates.[41–44] Methods include broth dilution, disc dilution, antifungal impregnated gradient strips and agar screening as described by European Committee on Antimicrobial Susceptibility Testing (EUCAST) or the Clinical & Laboratory Standards Institute (CSLI). Resistance may be intrinsic (e.g. resistance of *Candida krusei* to fluconazole) or an acquired trait following prolonged drug exposure. If resistance is uncommon (e.g. *Aspergillus fumigatus* to voriconazole), empiric treatment can be determined by accurately identifying the pathogen without routine susceptibility testing. In addition, if there is no interpretive data (breakpoint or ecological cut-off (ECOFF), the MIC alone may be of limited clinical value, and the benefit of routine susceptibility testing has not been determined. For isolates from systemic infection, there is a trend toward infection with non-*albicans* species of *Candida* and an increase in antifungal resistance,[45]and acquired resistance has emerged in

For peer review only

species such as *Candida glabrata* and *Candida auris* (rarely reported as an ocular pathogen). There has also been an increase in the resistance of *Aspergillus fumigatus* to azoles, especially in Europe,[46] linked to the use of azoles in agriculture.[47] However, polyenes (natamycin, amphotericin B) are preferred for empiric therapy of fungal keratitis rather than azoles.[48,49] Therapeutic guidelines for fungal keratitis have largely been developed from randomised controlled trials (RCTs) performed in South Asia.[48–50] Importantly, isolates from these studies were predominantly filamentous fungi. Comparisons of antifungal resistance between regions for isolates from keratitis have not been performed.

Speciation of isolates of *Acanthamoeba* spp. or susceptibility testing against biocides is not usually performed or discussed by the EUCAST or CSLI. There is a lack of consensus on the optimum protocol for testing trophozoites or cysts against antiseptics (polyhexanide, chlorhexidine).[51] When it is done, susceptibility testing is performed on cysts because most amoebicides readily kill trophozoites. The usual test is based on preventing excystment after culture, or assessing cyst viability by staining with trypan blue, following exposure to serial dilutions for one to seven days *in vitro*.[52,53] However, this protocol is primarily used for the assessment of potential amoebicidal drugs rather than for clinical management or audit of resistance. A few publications have used these methods to show a poor correlation between *in vitro* susceptibility and clinical eradication of acanthamoeba from the cornea.[54,55]

The goal of antimicrobial susceptibility testing is to reliably produce data that may be used to guide patient therapy, inform epidemiological studies, and track rates of AMR. AMR is measured by the response of a pathogen cultured *in vitro* in the presence of increasing concentrations of an antimicrobial agent. The critical measurement for antimicrobial susceptibility testing is the MIC - the concentration of the antimicrobial that inhibits overnight growth of the microbe cultured from the patient. This MIC is then referenced to a breakpoint, a

For peer review only - http://bmjopen.bmj.com/site/about/guidelines.xhtml

chosen antimicrobial concentration (mg/L) that defines the likelihood of clinical success for that agent against the microbe. The breakpoint is a tool to link a MIC value generated by *in vitro* testing to the most likely *in vivo* response of that isolate to an achievable concentration of the drug. If there is an established breakpoint, the MIC can then be used to differentiate isolates in which there is a high likelihood of treatment success from those in which treatment is more likely to fail. The breakpoint is determined from the MIC distribution of a well-characterised wild-type population, the pharmacokinetics of the antimicrobial *in vivo*, host status, and clinical trials, to identify the lowest MIC to give a good clinical outcome. Ideally, the MIC should be at the lower end of the achievable tissue concentration. However, *in vitro* resistance does not necessarily equate to treatment failure. The clinical breakpoints of topically applied antimicrobials are unknown, and values for topical treatment of corneal infection are based on achievable and safe serum concentrations that may not be relevant for topically applied antimicrobials. Suggested ophthalmic breakpoints for bacteria and fungi are provided by regulatory authorities such as EUCAST or CSLI.[56,57] For microbial keratitis, it is difficult to establish a breakpoint without outcome data, so defining the epidemiological cutoff value (ECV or ECOFF) is an alternative. The ECOFF is not equivalent to a breakpoint but is the MIC that separates isolates into those with or without acquired or mutational resistance by defining the upper limit of the wild-type MIC distribution. Again, it does not predict clinical success but indicates whether a specific isolate carries resistance to an antimicrobial that would otherwise be active against that species.

Several studies have reported AMR in isolates from microbial keratitis. A recent systematic review and meta-analysis estimated differences in the spectrum of bacterial isolates between regions, the overall prevalence of resistance, and changes over time.[5] However, there have been no reviews of resistance in either fungal or amoebic keratitis. The review by Zhang et al. was limited to citations in English published after 2000, with an emphasis on reporting positive culture rates and the spectrum of bacterial isolates globally, with no regional data.[5] We will

For peer review only

extend the study to all microbes, and include all publications with no limit to time or language. We will report differences in resistance between regions. Because the actual ophthalmic breakpoints are unknown, we will also record the MIC data where this is available. This is because reporting the prevalence of resistance does not identify the primary measurement, which is the MIC. In addition, changes in MIC can occur before the effect of this is evident as a change in resistance values (MIC drift). Establishing the prevalence of antimicrobial resistance may focus attention on the magnitude of the problem and serve as a benchmark for future publications.

Objectives

- To report the proportion of the common pathogens isolated from microbial keratitis that are resistant to the antimicrobials commonly used as empiric therapy, and report the distribution of their MICs.
- To explore how the proportion of AMR and the distribution of MICs varies by region.
- To explore if the proportion of AMR and the distribution of MICs in common pathogens has changed over time.

METHODS AND ANALYSIS

Protocol and registration

We report this protocol following the Preferred Reporting Items for Systematic Reviews and Meta-Analyses Protocols (PRISMA-P) guidelines.[58] The protocol has been registered <**PROSPERO registration number – registration after reviewer comments**>. Any protocol amendments will be documented, including the date of amendment and rationale for the change in the final review report.

Types of studies

We will include published prospective and retrospective cross-sectional studies that have identified the genus and species of isolates from corneal cultures and recorded the susceptibility or MIC of each isolate to eye-appropriate topical antimicrobials. We will also consider the baseline findings (pre-randomisation) of people recruited in clinical trials for treatment or cohort studies. We will only include peer-reviewed articles published in any language. We will not include grey literature or conference abstracts. We will consider publications from all years and geographical regions.

Types of participants, eligibility and setting

Inclusion criteria will be studies that report the investigation results for suspected microbial keratitis, including bacterial, fungal and acanthamoeba keratitis. We will exclude cases or studies that only report on viral isolates or *in vitro* resistance to antivirals. There will be no age restrictions. Settings will include primary care as well as secondary and tertiary referral centres. Studies must report the results of an investigation, including the identification of the organisms grown in culture (genus and species) and their anticipated susceptibility or resistance to antimicrobials, with the measured MIC if this is available. The genus and species will be recorded. The method of assessment (disc diffusion, Etest, tube dilution) will be recorded. The regulatory body that provided the assessment thresholds or breakpoints will be recorded, such as EUCAST, CSLI, ChiCAST. We will exclude studies where participants are exclusively patients in specific settings (ICU), or patients with specific syndromes (SJS) or other disease groups (non-healing ulcers, corneal graft for treatment failure). We will include studies irrespective of pre-treatment of patients. We will group the results as bacteria, fungi or acanthamoeba, and we will pool data from studies within regions. The absolute number of isolates and the proportion of the total isolates will be recorded. Differences between the method of identification (MALDI-TOF vs biochemical assay) for bacteria is not thought to be

significant. Results from PCR will not be included as these do not routinely measure AMR. We will not analyse the response to therapy.

Types of outcome measures

- The proportion of the common pathogens isolated from suspected microbial keratitis that are resistant to the antimicrobials commonly used as empiric therapy. Results will be recorded as sensitive (S), sensitive at an increased dosage (I), or resistant (R). For the analysis the S and I groups will be amalgamated.
- The MIC values (mg/L) for the antimicrobials commonly used as empiric therapy for suspected microbial keratitis. Results will be reported as the mode and range.

Search strategies

Electronic searches

We will conduct an electronic bibliographic search in MEDLINE, EMBASE, Web of Science, Cumulative Index to Nursing and Allied Health Literature (CINAHL), Google Scholar and the Cochrane Library. An expert information specialist developed the search strategies (Supplementary Figure 1). Relevant publications will be retrieved manually if electronic access is not available.

Searching for other resources

We will identify additional studies by searching the reference lists of relevant publications identified through electronic searches and prior review articles on this topic. We will consult trial registries such as ClinicalTrials.gov to identify studies indexed in the databases.

Selection of studies

Two review authors will independently screen search results based on title and abstract and will remove reports that do not fall into the scope of this review. We will resolve disagreements by discussion and consultation with another author as needed. We will acquire the full text of all

For peer review only - http://bmjopen.bmj.com/site/about/guidelines.xhtml

publications appearing to meet the criteria for inclusion in this review. Two review authors will independently screen the full-text reports using the inclusion criteria listed above. They will discuss any disagreements, and a third review author will arbitrate if they cannot resolve them. Screening of search results will be conducted using Covidence systematic review software (Veritas Health Innovation, Melbourne, Australia; available at www.covidence.org).

Data extraction and management

Data extraction will be done in Covidence using a customised data extraction template which will be pre-piloted on 5 studies (Table 1). Two review authors will independently extract the following data from each study: study design, participant characteristics, study population and size, study setting, study dates, diagnostic and ascertainment methods, details of pathogen and antimicrobials, number of isolates, the proportion of isolates reported resistant (R) for each antimicrobial, and the MIC values of these antimicrobials. We will document the method used for speciation of the organism, how the MIC was determined, and the reference guidelines for determining breakpoint thresholds. Disagreements will be resolved by discussion and consultation with another author as needed; a third review author will arbitrate if they cannot resolve them.

AMR will be recorded separately for bacteria, fungi and acanthamoeba. Within each phylum, the AMR will be reported for each class of antimicrobial against each genus of organism.

- For bacterial isolates, the genus and species of all isolates will be recorded, with a secondary grouping of pathogens into classes. The groups will be: *Enterobacterales, Pseudomonas* spp., *Staphylococcus* spp., *methicillin-resistant S. aureus* (MRSA), *coagulase-negative staphylococci (CNS)*, *Streptococcus pneumoniae,* viridans group streptococci *(VGS)*, *beta-haemolytic streptococci*, *Haemophilus* spp., *Neisseria* spp. The antimicrobial groups will be quinolones (second and subsequent generations), beta-

For peer review only

lactams (early and late cephalosporins), aminoglycosides, chloramphenicol, glycopeptides and antiseptics.

- For fungal isolates, the genus and species will be recorded, with a secondary grouping of the pathogens into classes. The groups will be yeast and filamentous fungi. The antifungal groups will be the polyenes (natamycin, amphotericin), azoles (miconazole, econazole, voriconazole), echinocandins (micafungin) and antiseptics.
- For amoeba isolates the genus will be recorded and the species if this has been done. The antiamoebic treatments include polyhexanide, chlorhexidine, voriconazole and neomycin.

Table 1. Data extraction domains for included studies

	Domain/subdomain	Description
1	***Document characteristics***	
	Title, authors, publication year	Title, authors, and year of publication
	Full citation and web link	Citation of publication and PMID
2	***Study characteristics***	
	Design	Prospective study, retrospective study, etc.
	Setting	Primary care, hospital eye service, etc.
	Location	City and country of data collection
	Data collection	Years when samples collected
	Population	Eligibility criteria, age range, specific risk group (LVC, SJS)
	Sample size	Number of participants in the study
	Study objectives	What was the study research question?
3	***Characteristics***	
	Inclusion criteria	Presumed microbial keratitis, size, position
	Exclusion criteria	Prior antimicrobial treatment, perforation

For peer review only

	Definition of significant isolate	Growth on two media, supportive investigation (PCR, IVCM)
	Microbial isolate	Bacteria, fungus, acanthamoeba
	Method of identification	e.g., MALDI-TOF, phenotypic and biochemical, molecular
	Determination of MIC	Tube dilution, Etest, disc diffusion. Effect on outcome recorded.
	Reference body	EUCAST, CSLI, ChiCAST
	Bacterial groups (s)	Enterobacteriales, Pseudomonas spp, Staphylococcus spp, MRSA, Staphylococcus (CNS), Streptococcus pneumonia, Streptococcus (VTS), beta-haemolytic Streptococcus, Haemophilus spp, Neisseria spp.
	Sensitivity and MIC of bacteria to antimicrobials	Quinolones, beta-lactam, aminoglycoside, glycopeptide, chloramphenicol, antiseptics
	Fungal group(s)	Yeast and filamentary fungi
	Sensitivity and MIC of fungi to antifungals	Polyenes, azoles, echinocandins, antiseptics
	Amoeba isolates	Acanthamoeba
	Sensitivity and MIC of amoeba to amebicides	Polyhexanide, chlorhexidine, voriconazole, neomycin

MIC minimum inhibitory concentration, EUCAST European Committee on Antimicrobial Susceptibility Testing, CSLI Clinical & Laboratory Standards Institute, MRSA methicillin resistant Staphylococcus aureus, VTS viridans-type streptococci, MALDI-TOF matrix-assisted laser desorption/ionization time of flight, LVC laser vision correction, SJS Stevens Johnson syndrome. PCR polymerase chain reaction, IVCM in vivo confocal microscopy

For peer review only

Assessment of risk of bias

Two reviewers will independently assess the risk of bias in each included study using the risk of bias tool for prevalence studies developed by Hoy et al.[59] This tool covers four domains - selection bias, non-response bias, measurement bias and bias related to analysis. For each of the 10 items included in the assessment, two reviewers will record the rationale for their judgements. The Risk of Bias (Supplementary Table 1) shows the items included in the tool and specific considerations for this review. This risk of bias tool will be piloted on 5 studies and instructions for reviewers amended in the light of that pilot. Agreement between reviewers will

be assessed and any disagreements resolved by discussion. For each study, a judgement of the overall risk of bias (low, moderate, or high) will be made.

Dealing with missing data

If we do not find all of the necessary information in a published study, for articles published in 2010 or later, we will email the corresponding author to solicit further information. If we cannot obtain the necessary information, we will document in the review that we attempted to contact the study authors. We will report and discuss the possible effect of missing data on each study and the overall review and meta-analysis. We will consider the susceptibility of our meta-analysis to the impact of missing data. We will analyse the data that is available rather than imputing missing data.

Unit of analysis issues

We will document how each study handled eyes - whether one eye or both eyes were included and analysed in the study. We will also document how the study handled multiple isolates per person or eye. Where multiple isolates are identified we will consider each isolate separately and analyse as reported. Where possible, we will extract proportions/confidence intervals adjusted for clustered data or consider adjusting using methods outlined in the Cochrane Handbook.[60]

Data synthesis and meta-analysis approach

We will perform meta-analyses using a random-effects model in Stata V.17 (StataCorp).[61] We will summarise and report the pooled proportion of resistance, its 95% CI, and between-study heterogeneity (I^2). We will conduct meta-analyses separately for each class of antimicrobial (Table 1): fluoroquinolones (second and fourth generation separately), beta-lactam antibiotics (cefuroxime), aminoglycosides (gentamicin, tobramycin), glycopeptide antibiotics (vancomycin) for bacterial; natamycin, voriconazole, amphotericin, (and others) for fungi (filamentous fungi,

For peer review only

yeasts); polyhexanide (PHMB) and voriconazole for *Acanthamoeba* spp. We will consider amalgamating closely related groups of organisms (Table 1) that have similar mechanisms of AMR.

We anticipate heterogeneity in reporting of MIC values and therefore we plan to report these data narratively in structured tables and figures. In the event that comparable figures on MIC in mg/L are available from different studies we will consider a meta-analysis as specified above.

Meta regression

If there are sufficient studies for analysis we will include the following covariates:

- Class of microbe: We intend to present data for the eight most frequent Gram-positive (*S. aureus*, *Streptococcus* spp. etc) and Gram-negative (*P. aeruginosa*, etc) isolates.
- Region: we will group studies according to the global super-region as defined by the Global Burden of Disease Study.[62]
- Decade: we will group studies according to the decade in which they were conducted: Before 2000, 2000-2009, 2010-2019, 2020 - 2022.
- Pre-treatment (none, some, all).
- Sex (M:F)
- Age (children:adults >16 years)
- Study size. We will assess small study effects, one of which may be publication bias, by preparing a funnel plot, which is a scatter plot of effect size versus precision (standard error).[63]

Assessment of heterogeneity

We will assess clinical heterogeneity by comparing key participant characteristics at the study level (e.g., age, sex, ocular diagnoses). Methodological heterogeneity will also be considered, including comparing the risk of bias of included studies. We will assess statistical heterogeneity

For peer review only

by inspecting forest plots and through the I^2 statistic. If high levels of inconsistency are detected, and there are enough studies, we will explore likely sources of this heterogeneity (see section on meta-regression).

Sensitivity analysis

We will conduct a sensitivity analysis of the primary analysis in which studies are excluded if they are judged to be at high risk of bias.

Assessing the certainty of the evidence

We will assess and report the overall certainty of evidence from our analyses and critical appraisal using the modified Grades of Recommendation, Assessment, Development and Evaluation tool.[64] We will consider evidence from cross-sectional studies to be high certainty and will downgrade for risk of bias, imprecision, inconsistency, indirectness and publication bias. The GRADE assessment will be done by consensus discussion.

Patient and public involvement statement

As we plan to review existing published literature only, this review will be performed without specific patient or public involvement.

Dissemination

Findings will be published in an open-access peer-reviewed journal and presented at national and international meetings. We anticipate that the findings will be of considerable interest to those involved in eye health provision, as well as the general medical, public health, development, and governmental sectors.

Strengths and limitations of this study

- This systematic review protocol follows the Preferred Reporting Items for Systematic Review and Meta-Analyses Protocols (PRISMA-P) guidelines.

For peer review only

- This systematic review addresses a gap in the current evidence by estimating the MIC and prevalence by region of AMR in common corneal pathogens.
- There may be significant clinical, methodological, and statistical heterogeneity in reporting prevalence data between different populations. A potential limitation might be the lack of published literature on MICs and the method of extrapolation of this data to the definition of clinical resistance.
- Further potential limitations are the application of clinical thresholds for sampling, and sampling of cases in a hospital setting that may not be representative of the full spectrum of microbial keratitis.

CONCLUSIONS

The aim of this systematic review and meta-analyses is to provide an overall estimate of the levels of AMR to first line antimicrobials in the classes of pathogens frequently isolated from microbial keratitis. The name of authors, region, study design, year of publication, sample size will be summarised. The data will be recorded using a structured data extraction form and presented using figures and tables. We believe the findings of this review will provide important evidence regarding the prevalence of AMR with associated MICs that will provide benchmark data. This will inform clinicians, clinical scientists, and stakeholders including policymakers, health service managers and service users.

Contributors

ST and JE drafted the protocol with suggestions from the other authors. IG developed the search strategy. ST is the guarantor. All authors read and approved the final draft.

Funding

For peer review only

MJB, AL and DM and supported by Wellcome Trust (207472/Z17/Z). The funder had no role in the development of the review.

Competing interests

None declared

Provenance and peer review

Not commissioned, externally peer reviewed

Data availability statement

No data is available.

Ethics Approval: Ethics approval is not required as this is a protocol for a systematic review of published data.

References

1 Brown L, Leck AK, Gichangi M, *et al.* The global incidence and diagnosis of fungal keratitis. *Lancet Infect Dis* 2021;**21**:e49–57.

2 Song X, Xie L, Tan X, *et al.* A multi-center, cross-sectional study on the burden of infectious keratitis in China. *PLoS One* 2014;**9**:e113843.

3 Stapleton F. The epidemiology of infectious keratitis. *Ocul Surf* Published Online First: 19 August 2021. doi:10.1016/j.jtos.2021.08.007

4 Erie JC, Nevitt MP, Hodge DO, *et al.* Incidence of ulcerative keratitis in a defined population from 1950 through 1988. *Arch Ophthalmol* 1993;**111**:1665–71.

5 Zhang Z, Cao K, Liu J, *et al.* Pathogens and Antibiotic Susceptibilities of Global Bacterial Keratitis: A Meta-Analysis. *Antibiotics (Basel)* 2022;**11**. doi:10.3390/antibiotics11020238

6 Lalitha P, Manoharan G, Karpagam R, *et al.* Trends in antibiotic resistance in bacterial keratitis isolates from South India. *Br J Ophthalmol* 2017;**101**:108–13.

7 Srinivasan M, Mascarenhas J, Rajaraman R, *et al.* Corticosteroids for bacterial keratitis: the Steroids for Corneal Ulcers Trial (SCUT). *Arch Ophthalmol* 2012;**130**:143–50.

8 Bharathi MJ, Ramakrishnan R, Vasu S, *et al.* Aetiological diagnosis of microbial keratitis in South India - a study of 1618 cases. *Indian J Med Microbiol* 2002;**20**:19–24.

9 Shah A, Sachdev A, Coggon D, *et al.* Geographic variations in microbial keratitis: an analysis of the peer-reviewed literature. *Br J Ophthalmol* 2011;**95**:762–7.

10 Lin A, Rhee MK, Akpek EK, *et al.* Bacterial Keratitis Preferred Practice Pattern®. *Ophthalmology* 2019;**126**:P1–55.

11 Ung L, Wang Y, Vangel M, *et al.* Validation of a Comprehensive Clinical Algorithm for the Assessment and Treatment of Microbial Keratitis. *Am J Ophthalmol* 2020;**214**:97–109.

12 Arunga S, Kintoki GM, Mwesigye J, *et al.* Epidemiology of Microbial Keratitis in Uganda: A Cohort Study. *Ophthalmic Epidemiol* 2020;**27**:121–31.

13 Blair JMA, Webber MA, Baylay AJ, *et al.* Molecular mechanisms of antibiotic resistance. *Nat Rev Microbiol* 2015;**13**:42–51.

14 Leibovitch I, Lai TF, Senarath L, *et al.* Infectious keratitis in South Australia: emerging resistance to cephazolin. *Eur J Ophthalmol* 2005;**15**:23–6.

15 Shalchi Z, Gurbaxani A, Baker M, *et al.* Antibiotic resistance in microbial keratitis: ten-year experience of corneal scrapes in the United Kingdom. *Ophthalmology* 2011;**118**:2161–5.

16 Alexandrakis G, Alfonso EC, Miller D. Shifting trends in bacterial keratitis in south Florida

For peer review only

and emerging resistance to fluoroquinolones. *Ophthalmology* 2000;**107**:1497–502.

17 Littmann J, Viens AM. The Ethical Significance of Antimicrobial Resistance. *Public Health Ethics* 2015;**8**:209–24.

18 Ray KJ, Prajna L, Srinivasan M, *et al.* Fluoroquinolone treatment and susceptibility of isolates from bacterial keratitis. *JAMA Ophthalmol* 2013;**131**:310–3.

19 Goldstein MH, Kowalski RP, Gordon YJ. Emerging fluoroquinolone resistance in bacterial keratitis: a 5-year review. *Ophthalmology* 1999;**106**:1313–8.

20 Garg P, Sharma S, Rao GN. Ciprofloxacin-resistant Pseudomonas keratitis. *Ophthalmology* 1999;**106**:1319–23.

21 Peng MY, Cevallos V, McLeod SD, *et al.* Bacterial Keratitis: Isolated Organisms and Antibiotic Resistance Patterns in San Francisco. *Cornea* 2018;**37**:84–7.

22 Ni N, Nam EM, Hammersmith KM, *et al.* Seasonal, geographic, and antimicrobial resistance patterns in microbial keratitis: 4-year experience in eastern Pennsylvania. *Cornea* 2015;**34**:296–302.

23 Kaliamurthy J, Kalavathy CM, Parmar P, *et al.* Spectrum of bacterial keratitis at a tertiary eye care centre in India. *Biomed Res Int* 2013;**2013**:181564.

24 Lichtinger A, Yeung SN, Kim P, *et al.* Shifting trends in bacterial keratitis in Toronto: an 11-year review. *Ophthalmology* 2012;**119**:1785–90.

25 Asbell PA, Sanfilippo CM, Sahm DF, *et al.* Trends in Antibiotic Resistance Among Ocular Microorganisms in the United States From 2009 to 2018. *JAMA Ophthalmol* 2020;**138**:439–50.

For peer review only

26 Lin L, Duan F, Yang Y, *et al.* Nine-year analysis of isolated pathogens and antibiotic susceptibilities of microbial keratitis from a large referral eye center in southern China. *Infect Drug Resist* 2019;**12**:1295–302.

27 Singh M, Gour A, Gandhi A, *et al.* Demographic details, risk factors, microbiological profile, and clinical outcomes of pediatric infectious keratitis cases in North India. *Indian J Ophthalmol* 2020;**68**:434–40.

28 Oldenburg CE, Lalitha P, Srinivasan M, *et al.* Emerging moxifloxacin resistance in Pseudomonas aeruginosa keratitis isolates in South India. *Ophthalmic Epidemiol* 2013;**20**:155–8.

29 Chang VS, Dhaliwal DK, Raju L, *et al.* Antibiotic Resistance in the Treatment of Staphylococcus aureus Keratitis: a 20-Year Review. *Cornea* 2015;**34**:698–703.

30 Cariello AJ, Passos RM, Yu MCZ, *et al.* Microbial keratitis at a referral center in Brazil. *Int Ophthalmol* 2011;**31**:197–204.

31 Acharya M, Farooqui J, Gaba T, *et al.* Delhi infectious keratitis study: Update on clinico-Microbiological profile and outcomes of infectious keratitis. Journal of Current Ophthalmology. 2020;**32**:249. doi:10.4103/joco.joco_113_20

32 Hernandez-Camarena JC, Graue-Hernandez EO, Ortiz-Casas M, *et al.* Trends in Microbiological and Antibiotic Sensitivity Patterns in Infectious Keratitis. Cornea. 2015;**34**:778–85. doi:10.1097/ico.0000000000000428

33 Vola ME, Moriyama AS, Lisboa R, *et al.* Prevalence and antibiotic susceptibility of methicillin-resistant Staphylococcus aureus in ocular infections. *Arq Bras Oftalmol* 2013;**76**:350–3.

For peer review only

34 Magiorakos A-P, Srinivasan A, Carey RB, *et al.* Multidrug-resistant, extensively drug-resistant and pandrug-resistant bacteria: an international expert proposal for interim standard definitions for acquired resistance. *Clin Microbiol Infect* 2012;**18**:268–81.

35 Tacconelli E, Carrara E, Savoldi A, *et al.* Discovery, research, and development of new antibiotics: the WHO priority list of antibiotic-resistant bacteria and tuberculosis. *Lancet Infect Dis* 2018;**18**:318–27.

36 Fernandes M, Vira D, Medikonda R, *et al.* Extensively and pan-drug resistant Pseudomonas aeruginosa keratitis: clinical features, risk factors, and outcome. *Graefes Arch Clin Exp Ophthalmol* 2016;**254**:315–22.

37 Vazirani J, Wurity S, Ali MH. Multidrug-Resistant Pseudomonas aeruginosa Keratitis: Risk Factors, Clinical Characteristics, and Outcomes. *Ophthalmology* 2015;**122**:2110–4.

38 Berkow EL, Lockhart SR, Ostrosky-Zeichner L. Antifungal Susceptibility Testing: Current Approaches. *Clin Microbiol Rev* 2020;**33**. doi:10.1128/CMR.00069-19

39 Odds FC, Brown AJP, Gow NAR. Antifungal agents: mechanisms of action. *Trends Microbiol* 2003;**11**:272–9.

40 Perlin DS, Rautemaa-Richardson R, Alastruey-Izquierdo A. The global problem of antifungal resistance: prevalence, mechanisms, and management. *Lancet Infect Dis* 2017;**17**:e383–92.

41 Oechsler RA, Feilmeier MR, Miller D, *et al.* Fusarium keratitis: genotyping, in vitro susceptibility and clinical outcomes. *Cornea* 2013;**32**:667–73.

42 Lalitha P, Sun CQ, Prajna NV, *et al.* In vitro susceptibility of filamentous fungal isolates from a corneal ulcer clinical trial. *Am J Ophthalmol* 2014;**157**:318–26.

For peer review only

43 Mukherjee PK, Chandra J, Yu C, *et al.* Characterization of fusarium keratitis outbreak isolates: contribution of biofilms to antimicrobial resistance and pathogenesis. *Invest Ophthalmol Vis Sci* 2012;**53**:4450–7.

44 Kimura K, Inoue Y, Asari S, *et al.* Multicenter prospective observational study of fungal keratitis in Japan: analyses of in vitro susceptibility tests for combinations of drugs. *Jpn J Ophthalmol* 2022;**66**:240–53.

45 Gonçalves SS, Souza ACR, Chowdhary A, *et al.* Epidemiology and molecular mechanisms of antifungal resistance in Candida and Aspergillus. *Mycoses* 2016;**59**:198–219.

46 Resendiz Sharpe A, Lagrou K, Meis JF, *et al.* Triazole resistance surveillance in Aspergillus fumigatus. *Med Mycol* 2018;**56**:83–92.

47 Chowdhary A, Kathuria S, Xu J, *et al.* Correction: Emergence of Azole-Resistant Aspergillus fumigatus Strains due to Agricultural Azole Use Creates an Increasing Threat to Human Health. PLoS Pathogens. 2013;**9**. doi:10.1371/annotation/4ffcf1da-b180-4149-834c-9c723c5dbf9b

48 Hoffman JJ, Yadav R, Sanyam SD, *et al.* Topical Chlorhexidine 0.2% versus Topical Natamycin 5% for the Treatment of Fungal Keratitis in Nepal: A Randomized Controlled Noninferiority Trial. *Ophthalmology* Published Online First: 9 December 2021. doi:10.1016/j.ophtha.2021.12.004

49 Prajna NV, Krishnan T, Mascarenhas J, *et al.* The mycotic ulcer treatment trial: a randomized trial comparing natamycin vs voriconazole. *JAMA Ophthalmol* 2013;**131**:422–9.

50 Prajna NV, Krishnan T, Rajaraman R, *et al.* Effect of Oral Voriconazole on Fungal Keratitis in the Mycotic Ulcer Treatment Trial II (MUTT II): A Randomized Clinical Trial. *JAMA Ophthalmol* 2016;**134**:1365–72.

For peer review only

51 Shing B, Balen M, McKerrow JH, *et al.* Acanthamoeba Keratitis: an update on amebicidal and cysticidal drug screening methodologies and potential treatment with azole drugs. *Expert Rev Anti Infect Ther* 2021;**19**:1427–41.

52 Narasimhan S, Madhavan HN, K LT. Development and application of an in vitro susceptibility test for Acanthamoeba species isolated from keratitis to polyhexamethylene biguanide and chlorhexidine. *Cornea* 2002;**21**:203–5.

53 Turner NA, Russell AD, Furr JR, *et al.* Emergence of resistance to biocides during differentiation of Acanthamoeba castellanii. *J Antimicrob Chemother* 2000;**46**:27–34.

54 Elder MJ, Kilvington S, Dart JK. A clinicopathologic study of in vitro sensitivity testing and Acanthamoeba keratitis. *Invest Ophthalmol Vis Sci* 1994;**35**:1059–64.

55 Pérez-Santonja JJ, Kilvington S, Hughes R, *et al.* Persistently culture positive acanthamoeba keratitis: in vivo resistance and in vitro sensitivity. *Ophthalmology* 2003;**110**:1593–600.

56 Clinical & Laboratory Standards Institute: CLSI guidelines. Clinical & Laboratory Standards Institute. https://clsi.org/ (accessed 18 Mar 2022).

57 ESCMID-European Society of Clinical Microbiology, Diseases I. EUCAST: EUCAST. https://eucast.org/ (accessed 18 Mar 2022).

58 Moher D, Shamseer L, Clarke M, *et al.* Preferred reporting items for systematic review and meta-analysis protocols (PRISMA-P) 2015 statement. *Syst Rev* 2015;**4**:1.

59 Hoy D, Brooks P, Woolf A, *et al.* Assessing risk of bias in prevalence studies: modification of an existing tool and evidence of interrater agreement. *J Clin Epidemiol* 2012;**65**:934–9.

60 Higgins JPT, Eldridge S, Li T. Including variants on randomized trials. Cochrane Handbook

For peer review only

for Systematic Reviews of Interventions. 2019;:569–93. doi:10.1002/9781119536604.ch23

61 Nyaga VN, Arbyn M, Aerts M. Metaprop: a Stata command to perform meta-analysis of binomial data. *Arch Public Health* 2014;**72**:39.

62 GBD regions and super regions. The International Agency for the Prevention of Blindness. 2020.https://www.iapb.org:8443/learn/vision-atlas/about/definitions-and-regions/ (accessed 23 Feb 2022).

63 Egger M, Davey Smith G, Schneider M, *et al.* Bias in meta-analysis detected by a simple, graphical test. *BMJ* 1997;**315**:629–34.

64 Iorio A, Spencer FA, Falavigna M, *et al.* Use of GRADE for assessment of evidence about prognosis: rating confidence in estimates of event rates in broad categories of patients. *BMJ* 2015;**350**:h870.

For peer review only

Search Strategy

MEDLINE

1. keratitis/ or acanthamoeba keratitis/
2. keratitis.tw.
3. (fung$ adj3 infect$ adj3 eye$).tw.
4. (fung$ adj3 infect$ adj3 ocular).tw.
5. (bacteria$ adj3 infect$ adj3 eye$).tw.
6. (bacteria$ adj3 infect$ adj3 ocular).tw.
7. or/1-6
8. exp Drug Resistance, Microbial/
9. ((antimicrobial or antifungal or increase$) adj2 resistan$).tw.
10. "AMR".tw.
11. Microbial Sensitivity Tests/
12. (drug adj2 (resistan$ or suscept$)).tw.
13. (minimum adj1 inhibit$ adj1 concentration$).tw.
14. "MIC".tw.
15. breakpoint.tw.
16. or/8-15
17. prevalence/
18. prevalence.tw.
19. case-control studies/ or retrospective studies/ or cohort studies/ or follow-up studies/ or longitudinal studies/ or prospective studies/ or cross-sectional studies/ or observational study/
20. (case adj1 control$ adj3 (stud$ or trial$)).tw.
21. (cross adj1 sectional$ adj3 (stud$ or trial$)).tw.
22. (follow adj1 up adj3 (stud$ or trial$)).tw.
23. ((prospective or retrospective or cohort or longitudinal or observational) adj3 (stud$ or trial$)).tw.
24. clinical trial/ or control groups/ or double-blind method/ or meta-analysis as topic/ or network meta-analysis/ or random allocation/ or single-blind method/
25. randomized controlled trial/ or controlled clinical trials as topic/ or randomized controlled trials as topic/ or comparative study/ or evaluation studies/ or meta- analysis/ or "systematic review"/
26. random$.tw.
27. or/17-26
28. 7 and 16 and 27

29 (animal or animals or mouse or mice or rat or rats or rabbit$ or dog or dogs or canine or cat or cats or pig or pigs or horse or horses or veterinary).tw.

30 28 not 29

Embase

1. keratitis/ or bacterial keratitis/ or virus keratitis/ or Acanthamoeba keratitis/ or microbial keratitis/ or suppurative keratitis/ or amebic keratitis/
2. keratitis.tw.
3. (fung$ adj3 infect$ adj3 eye$).tw.
4. (fung$ adj3 infect$ adj3 ocular).tw.
5. (bacteria$ adj3 infect$ adj3 eye$).tw.
6. (bacteria$ adj3 infect$ adj3 ocular).tw.
7. or/1-6
8. antibiotic resistance/ or antibiotic sensitivity/ or drug resistance/
9. aminoglycoside resistance/ or beta-lactam resistance/ or chloramphenicol resistance/ or fluoroquinolone resistance/
10. ((antimicrobial or antifungal or increase$) adj2 resistan$).tw.

For peer review only

11. "AMR".tw.
12. microbial sensitivity test/
13. (drug adj2 (resistan$ or suscept$)).tw.
14. minimum inhibitory concentration/
15. (minimum adj1 inhibit$ adj1 concentration$).tw.
16. "MIC".tw.
17. breakpoint.tw.
18. or/8-17
19. prevalence/
20. prevalence.tw.
21. controlled clinical trial/
22. case control study/
23. cohort analysis/
24. follow up/
25. longitudinal study/
26. observational study/
27. prospective study/
28. retrospective study/
29. cross-sectional study/
30. control group/
31. "meta analysis (topic)"/
32. network meta-analysis/
33. randomization/
34. single blind procedure/
35. double blind procedure/
36. control group/
37. controlled clinical trial/
38. clinical study/
39. clinical trial/
40. "clinical trial (topic)"/
41. "controlled clinical trial (topic)"/
42. "randomized controlled trial (topic)"/
43. comparative study/
44. evaluation study/
45. randomized controlled trial/
46. meta analysis/
47. "systematic review"/
48. "systematic review (topic)"/
49. (case adj1 control$ adj3 (stud$ or trial$)).tw.
50. (cross adj1 sectional$ adj3 (stud$ or trial$)).tw.
51. (follow adj1 up adj3 (stud$ or trial$)).tw.
52. ((prospective or retrospective or cohort or longitudinal or observational) adj3 (stud$ or trial$)).tw.
53. random$.tw.
54. or/19-53
55. 7 and 18 and 54
56. (animal or animals or mouse or mice or rat or rats or rabbit$ or dog or dogs or canine or cat or cats or pig or pigs or horse or horses or veterinary).tw.
57. 55 not 56

Cochrane Library

#1 MeSH descriptor: [Keratitis] this term only
#2 MeSH descriptor: [Acanthamoeba Keratitis] this term only
#3 keratitis
#4 fung* NEAR/3 infect* NEAR/3 eye
#5 fung* NEAR/3 infect* NEAR/3 ocular
#6 bacteria* NEAR/3 infect* NEAR/3 eye*

#7 bacteria* NEAR/3 infect* NEAR/3 ocular
#8 #1 or #2 or #3 or #4 or #5 or #6 or #7
#9 MeSH descriptor: [Drug Resistance, Microbial] explode all trees
#10 (antimicrobial or antifungal or increase*) NEAR/2 resistan*
#11 AMR
#12 MeSH descriptor: [Microbial Sensitivity Tests] this term only
#13 drug NEAR/2 (resistan* or suscept*)
#14 minimum NEXT inhibit* NEXT concentration*
#15 MIC
#16 breakpoint
#17 #9 or #10 or #11 or #12 or #13 or #14 or #15 or #16
#18 #8 and #17

Web of Science

#19 not #20
TS= (animal OR animals OR mouse OR mice OR rabbit* OR dog OR dogs OR canine OR horse OR horses OR veterinary)
#6 AND #11 AND #18
#17 OR #16 OR #15 OR #14 OR #13 OR #12
TS= (clinical OR comparative NEAR/3 (stud* OR trial*))
TS= (random* OR meta NEAR/1 analysis OR systematic NEAR/1 review)
TS= (follow NEAR/2 up NEAR/3 (stud* OR trial*))
TS= (cross NEAR/2 sectional* NEAR/3 (stud* OR trial*))
TS= (case NEAR/2 control* NEAR/3 (stud* OR trial*))
TS= (prevalence OR retrospective OR prospective OR cohort OR observational OR longitudinal)
#7 OR #8 OR #9 OR #10
TS= (drug NEAR/2 (resistan* or suscept*))
TS= ((antimicrobial OR antifungal OR increase*) NEAR/2 resistan*)
TS=(Microbial Sensitivity Tests)
TS=(Microbial Drug Resistance)
#1 OR #2 OR #3 OR #4 OR #5
TS= keratitis
TS= (fung* NEAR/3 infect* NEAR/3 eye*)
TS= (fung* NEAR/3 infect* NEAR/3 ocular)
TS= (bacteria* NEAR/3 infect* NEAR/3 eye*)
TS= (bacteria* NEAR/3 infect* NEAR/3 ocular)

Clinicaltrials.gov

(antimicrobial OR antifungal OR antibiotic OR drug) AND resistance | Keratitis

WHO ICTRP

Keratitis AND antimicrobial AND resistance OR Keratitis AND antifungal AND resistance
OR Keratitis AND antibiotic AND resistance OR Keratitis AND drug AND resistance

For peer review only

Supplementary Table 1: Risk of Bias Assessment

	Risk of bias item	Criteria for answers
1	Was the study's target population a close representation of the national population in relation to relevant variables?	Yes (LOW RISK)<br>The study's target population was a close representation of the national or regional population *e.g., population-based study or sampling of cases of microbial keratitis from representative settings.*<br>No (HIGH RISK)<br>The study's target population was clearly not representative of the national population. *e.g., sampling of cases attending tertiary referral center or cases selected from a population that had failed to improve with prior therapy with antimicrobials, selection of larger ulcers, exclusions of small peripheral ulcers or of impending or actual corneal perforation.*
2	Was the sampling frame a true or close representation of the target population?	Yes (LOW RISK): The sampling frame was a true or close representation of the target population, *e.g., an acceptable and standardized definition of microbial keratitis was used, including definitions of pathogens, standardized criteria for definition and selection of isolate type, and definition of significant growth.*<br><br>No (HIGH RISK): The sampling frame was NOT a true or close representation of the target population, e.g., *potential 'non-pathogens' were excluded. Unclear the basis for the clinical diagnosis. Definitions of pathogens and isolates are not standardized.*
3	Was some form of random selection used to select the sample, OR was a census undertaken?	Yes (LOW RISK): A census was undertaken, OR, some form of random selection was used to select the sample (e.g. simple random sampling, stratified random sampling, cluster sampling, systematic sampling), e.g. *random sample of a consecutive series of patients, or inclusion of a consecutive series of patients*<br><br>No (HIGH RISK): A census was NOT undertaken, AND some form of random selection was NOT used to select the sample *eg unclear how cases selected or unclear if consecutive or not*
4	Was the likelihood of non-response bias minimal?	Yes (LOW RISK): The response rate for the study was ≥75%, OR, an analysis was performed that showed no

For peer review only

		significant difference in relevant demographic characteristics between responders and nonresponders<br><br>No (HIGH RISK): The response rate was <75%, and if any analysis comparing responders and non-responders was done, it showed a significant difference in relevant demographic characteristics between responders and non-responders.
5	Were data collected directly from the subjects (as opposed to a proxy)?	Yes (LOW RISK): All data were collected directly from the subjects. *It is likely that data will be collected directly from all participants so this item may not be relevant and will be marked low risk of bias for all studies.*<br><br>No (HIGH RISK): In some instances, data were collected from a proxy. *Difficult to envisage a study where this would be the case.*
6	Was an acceptable and standardized case definition for AMR used in the study?	Yes (LOW RISK): An acceptable case definition was used, e.g., *definition of AMR clearly described and standardized (between geographical locations and over time if applicable)*<br><br>No (HIGH RISK): An acceptable case definition was NOT used. *e.g., unclear definition of AMR and/or definition not consistently applied in different groups of patients or over time.*
7	Was the study instrument that measured the parameter of interest shown to have validity and reliability?	Yes (LOW RISK): The study instrument had been shown to have reliability and validity (if this was necessary), e.g., test-retest, piloting, validation in a previous study, etc., *e.g., method of measuring AMR was valid and reliable i.e., valid laboratory systems for the identification of isolates and measurement of AMR or MIC.*<br><br>No (HIGH RISK): The study instrument had NOT been shown to have reliability or validity (if this was necessary), *e.g., method of measuring AMR was not clearly described or not validated.*
8	Was the same mode of data collection used for all subjects?	Yes (LOW RISK): The same mode of data collection was used for all subjects, e.g., *collection methodologies for the identified cases and measurement of resistance or MIC were adequately described and consistent during the course of the study.*

		No (HIGH RISK): The same mode of data collection was NOT used for all subjects, e.g., *collection methodologies for the identified and measurement of resistance or MIC not clearly described and/or inconsistent during the course of the study.*
9	Was the length of the shortest prevalence period for the parameter of interest appropriate?	Yes (LOW RISK): The shortest prevalence period for the parameter of interest was appropriate (e.g., point prevalence, one-week prevalence, one-year prevalence), e.g., *the study was done over an appropriate time period*<br><br>No (HIGH RISK): The shortest prevalence period for the parameter of interest was not appropriate (e.g., lifetime prevalence), e.g., *there may be seasonal variation in the proportions of isolates if the sampling interval was less than 12 months.*
10	Were the numerator(s) and denominator(s) for the parameter of interest appropriate?	Yes (LOW RISK): The paper presented appropriate numerator(s) AND denominator(s) for the parameter of interest, e.g., *it is clear that numerators and denominators are correct including grouping of species and documentation of potential pathogens/contaminants consistent in both groups.*<br><br>No (HIGH RISK): The paper did present numerator(s) AND denominator(s) for the parameter of interest but one or more of these were inappropriate
	Summary risk of bias	LOW RISK OF BIAS: Further research is very unlikely to change our confidence in the estimate.<br><br>MODERATE RISK OF BIAS: Further research is likely to have an important impact on our confidence in the estimate and may change the estimate.<br><br>HIGH RISK OF BIAS: Further research is very likely to have an important impact on our confidence in the estimate and is likely to change the estimate.

Adapted from Hoy et al.(Hoy et al. 2012)

Hoy, Damian, Peter Brooks, Anthony Woolf, Fiona Blyth, Lyn March, Chris Bain, Peter Baker, Emma Smith, and Rachelle Buchbinder. 2012. "Assessing Risk of Bias in Prevalence Studies: Modification of an Existing Tool and Evidence of Interrater Agreement." *Journal of Clinical Epidemiology* 65 (9): 934–39.

For peer review only

PRISMA-P 2015 Checklist

This checklist has been adapted for use with protocol submissions to *Systematic Reviews* from Table 3 in Moher D et al: Preferred reporting items for systematic review and meta-analysis protocols (PRISMA-P) 2015 statement. *Systematic Reviews* 2015 **4**:1

Section/topic	#	Checklist item	Information reported		Line number(s)
			Yes	No	
**ADMINISTRATIVE INFORMATION**					
**Title**					
Identification	1a	Identify the report as a protocol of a systematic review	☒	☐	Page 1
Update	1b	If the protocol is for an update of a previous systematic review, identify as such	☐	☒	
**Registration**	2	If registered, provide the name of the registry (e.g., PROSPERO) and registration number in the Abstract	☐	☒	Page 8
**Authors**					
Contact	3a	Provide name, institutional affiliation, and e-mail address of all protocol authors; provide physical mailing address of corresponding author	☒	☐	Page 1
Contributions	3b	Describe contributions of protocol authors and identify the guarantor of the review	☒	☐	Page 17
**Amendments**	4	If the protocol represents an amendment of a previously completed or published protocol, identify as such and list changes; otherwise, state plan for documenting important protocol amendments	☐	☒	
**Support**					
Sources	5a	Indicate sources of financial or other support for the review	☒	☐	Page 17
Sponsor	5b	Provide name for the review funder and/or sponsor	☒	☐	Page 17-18
Role of sponsor/funder	5c	Describe roles of funder(s), sponsor(s), and/or institution(s), if any, in developing the protocol	☐	☐	Page 17-18
**INTRODUCTION**					
**Rationale**	6	Describe the rationale for the review in the context of what is already known	☒	☐	Page 3-8
**Objectives**	7	Provide an explicit statement of the question(s) the review will address with reference to participants, interventions, comparators, and outcomes (PICO)	☒	☐	Page 8
**METHODS**					

BioMed Central
The Open Access Publisher

Section/topic	#	Checklist item	Information reported Yes	No	Line number(s)
**Eligibility criteria**	8	Specify the study characteristics (e.g., PICO, study design, setting, time frame) and report characteristics (e.g., years considered, language, publication status) to be used as criteria for eligibility for the review	☒	☐	Page 9
**Information sources**	9	Describe all intended information sources (e.g., electronic databases, contact with study authors, trial registers, or other grey literature sources) with planned dates of coverage	☒	☐	Page 10
**Search strategy**	10	Present draft of search strategy to be used for at least one electronic database, including planned limits, such that it could be repeated	☒	☐	Page 10
STUDY RECORDS					
Data management	11a	Describe the mechanism(s) that will be used to manage records and data throughout the review	☒	☐	Page 11
Selection process	11b	State the process that will be used for selecting studies (e.g., two independent reviewers) through each phase of the review (i.e., screening, eligibility, and inclusion in meta-analysis)	☒	☐	Page 11
Data collection process	11c	Describe planned method of extracting data from reports (e.g., piloting forms, done independently, in duplicate), any processes for obtaining and confirming data from investigators	☒	☐	Page 11
**Data items**	12	List and define all variables for which data will be sought (e.g., PICO items, funding sources), any pre-planned data assumptions and simplifications	☒	☐	Pages 11-12
**Outcomes and prioritization**	13	List and define all outcomes for which data will be sought, including prioritization of main and additional outcomes, with rationale	☒	☐	Pages 11-12
**Risk of bias in individual studies**	14	Describe anticipated methods for assessing risk of bias of individual studies, including whether this will be done at the outcome or study level, or both; state how this information will be used in data synthesis	☒	☐	Page 13
DATA					
**Synthesis**	15a	Describe criteria under which study data will be quantitatively synthesized	☒	☐	Page 14
	15b	If data are appropriate for quantitative synthesis, describe planned summary measures, methods of handling data, and methods of combining data from studies, including any planned exploration of consistency (e.g., I^2, Kendall's tau)	☒	☐	Pages 14-15
	15c	Describe any proposed additional analyses (e.g., sensitivity or subgroup analyses, meta-regression)	☒	☐	Page 15
	15d	If quantitative synthesis is not appropriate, describe the type of summary planned	☒	☐	Page 16
**Meta-bias(es)**	16	Specify any planned assessment of meta-bias(es) (e.g., publication bias across studies, selective reporting within studies)	☒	☐	Page 16
**Confidence in cumulative evidence**	17	Describe how the strength of the body of evidence will be assessed (e.g., GRADE)	☒	☐	Page 16

BioMed Central The Open Access Publisher

BMJ Open

Antimicrobial resistance in topical treatments for microbial keratitis: protocol for a systematic review and meta-analysis

Journal:	*BMJ Open*
Manuscript ID	bmjopen-2022-069338.R1
Article Type:	Protocol
Date Submitted by the Author:	02-Feb-2023

Complete List of Authors:	Tuft, Stephen; Moorfields Eye Hospital NHS Foundation Trust; Joint Library of Ophthalmology Moorfields Eye Hospital and UCL Institute of Ophthalmology<br>Evans, Jennifer; LSHTM, Faculty of Infectious and Tropical Diseases<br>Gordon, Iris; LSHTM, Department of Infectious and Tropical Diseases<br>Leck, Astrid; LSHTM, Department of Infectious and Tropical Diseases<br>Stone, Neil; University College London Hospitals NHS Foundation Trust, Department of Clinical Microbiology<br>Neal, Timothy; Royal Liverpool University Hospital<br>Macleod, David; LSHTM, Department of Medical Statistics<br>Kaye, Stephen; University of Liverpool<br>Burton, Matthew J; LSHTM, Faculty of Infectious and Tropical Diseases

**Primary Subject Heading**:	Ophthalmology
Secondary Subject Heading:	Infectious diseases, Epidemiology
Keywords:	MICROBIOLOGY, Corneal and external diseases < OPHTHALMOLOGY, EPIDEMIOLOGY, Public health < INFECTIOUS DISEASES

SCHOLARONE™ Manuscripts

Antimicrobial resistance in topical treatments for microbial keratitis: protocol for a systematic review and meta-analysis

Stephen Tuft[1,2], Jennifer R Evans[3,4], Iris Gordon[3], Astrid Leck[3], Neil Stone[5], Timothy Neal[6], David Macleod[3,7], Stephen Kaye[8], Matthew J Burton[1,3]

[1]Moorfields Eye Hospital, 162 City Road, London EC1V 2PD

[2]UCL Institute of Ophthalmology, 11-43 Bath Street, London EC1V 9EL

[3]International Centre for Eye Health, London School of Hygiene & Tropical Medicine, Keppel St, London WC1E 7HT

[4]Centre for Public Health, Queen's University Belfast, Institute of Clinical Science, Block A, Royal Victoria Hospital, Belfast BT12 6BA

[5]Department of Clinical Microbiology, University College London Hospitals NHS Foundation Trust, 250 Euston Road, London NW1 2PG.

[6]Department of Clinical Microbiology, Liverpool Clinical Laboratories, Liverpool University Hospital NHS Foundation Trust, Prescot Street, Liverpool L7 8XP.

[7]MRC International Statistics and Epidemiology Group, London School of Hygiene & Tropical Medicine, Keppel St, London WC1E 7HT

[8]Department of Eye and Vision Sciences, University of Liverpool, 6 West Derby Street, Liverpool L7 8TX.

Correspondence to:

Professor Stephen Tuft

Email: s.tuft@ucl.ac.uk

Word count: 3980

For peer review only

Keywords: Cornea, bacterial keratitis, fungus, acanthamoeba, microbial resistance, systematic review

ABSTRACT

Introduction: There is evidence for increased resistance against the antimicrobials used to treat keratitis. This review aims to provide global and regional prevalence estimates of antimicrobial resistance in corneal isolates, and the range of minimum inhibitory concentrations (MIC) with their associated resistance breakpoints.

Methods and analysis: We report this protocol following PRISMA-P guidelines. We will conduct an electronic bibliographic search in MEDLINE, EMBASE, Web of Science, and the Cochrane Library. Eligible studies will report in any language data for the resistance or MIC for antimicrobials against bacterial, fungal, or amoebic organisms isolated from suspected microbial keratitis. Studies that only report on viral keratitis will not be included. There will be no time restrictions on the date of publication. Screening for eligible studies, assessment of risk of bias and data extraction will be conducted by two reviewers independently, using predefined inclusion criteria and pre-piloted data extraction forms. We will resolve disagreements between the reviewers by discussion and, if required, a third (senior) reviewer will arbitrate. We will assess the risk of bias using a tool validated in prevalence studies. The certainty of the evidence will be assessed using the Grades of Recommendation, Assessment, Development and Evaluation (GRADE) approach. Pooled proportion estimates will be calculated using a random-effects model. Heterogeneity will be assessed using the I^2 statistic. We will explore differences between Global Burden of Disease regions and temporal trends.

Ethics approval and dissemination: Ethics approval is not required as this is a protocol for a systematic review of published data. The findings of this review will be published in an open-access, peer-reviewed journal.

PROSPERO registration number: CRD42023331126.

Strengths and limitations of this study

- This systematic review protocol follows the Preferred Reporting Items for Systematic Review and Meta-Analyses Protocols (PRISMA-P) guidelines.
- This systematic review addresses a gap in the current evidence by estimating the prevalence of antimicrobial resistance and minimum inhibitory concentrations (MICs) by Global Burden of Disease region in common corneal pathogens.
- There may be significant clinical, methodological, and statistical heterogeneity in reporting prevalence data between different populations.
- A potential limitation might be the lack of published literature on MICs and the method of extrapolation of this data to the definition of clinical resistance.
- Further potential limitations are the application of clinical thresholds for sampling, and sampling of cases in a hospital setting that may not be representative of the full spectrum of microbial keratitis.

INTRODUCTION

Microbial keratitis (MK) is a common corneal infection treated with topical antimicrobials. The causative organisms include bacteria, fungi, protozoa, and viruses, either singly or in combination. The proportions of these pathogens (viruses, bacteria, parasites, fungi) vary widely

between geographic regions, with fungal keratitis particularly prevalent in low- and middle-income countries and equatorial areas.[1] Published figures for the incidence of MK include 113 per 100,000 person-years in South India[1–3] and 799 per 100,000 person-years from Nepal.[4] A population-based study in China estimated the prevalence of past or active microbial keratitis to be 192 (95% CI 171–213) per 100,000, with a prevalence of presumed viral keratitis of 110, bacterial keratitis 75, and fungal keratitis 7 per 100,000.[2] The incidence figures for MK reported from high-income countries since 1995 are much lower with estimates of 4.5-37.7 cases per 100,000 person-years in the US, UK, Australia and Taiwan,[3] which follows a marked increase in cases from the 1970s onward associated with the widespread introduction of contact lens wear.[4]

The relative frequency and distribution of the potential bacterial pathogens isolated from corneal ulcers are usually derived from retrospective data rather than prospective surveys. Ideally, all ulcers should be sampled, as defining a threshold ulcer size to justify corneal culture will introduce case selection bias that could affect the spectrum of isolates.[5,6] The proportions vary widely between reports and according to the definition used for a significant isolate. In a recent meta-analysis of 38 studies, the most common isolates were *Staphylococcus* spp. (including *Staphylococcus aureus* and coagulase-negative staphylococci) (41.4%, 95% confidence limits 36.2–46.7%), *Pseudomonas* spp. (17.0%, 13.9–20.7%), *Streptococcus* spp. (13.1%, 10.9–15.7%), *Corynebacterium* spp. (6.6%, 5.3–8.3%) and *Moraxella* spp. *(4.1%, 3.1–5.4%).[7] Streptococcus pneumoniae* and *Nocardia* spp. are more frequently reported in series from South India.[8–10] Differences in environmental temperature, humidity, occupation, associated viral disease and malnutrition affect the local prevalence pattern.[11] In low-income countries, agricultural trauma is the major risk for infection due to filamentous fungi and *Acanthamoeba* spp., as opposed to contact lens wear in high-income regions.[12]

For peer review only

The goal of antimicrobial susceptibility testing is to reliably produce data that may be used to guide patient therapy, inform epidemiological studies, and track rates of antimicrobial resistance (AMR). AMR is measured by the response of a pathogen cultured *in vitro* in the presence of increasing concentrations of an antimicrobial agent. The critical measurement for antimicrobial susceptibility testing is the minimum inhibitory concentration (MIC) - the concentration of the antimicrobial that inhibits overnight growth of the microbe cultured from the patient. This MIC is then referenced to a breakpoint, a chosen antimicrobial concentration (mg/L) that defines the likelihood of clinical success for that agent against the microbe. The breakpoint is a tool to link a MIC value generated by *in vitro* testing to the most likely *in vivo* response of that isolate to an achievable drug concentration. If there is an established breakpoint, the MIC can then be used to differentiate isolates in which there is a high likelihood of treatment success from those in which treatment is more likely to fail. The breakpoint is determined from the MIC distribution of a well-characterised wild-type population, the pharmacokinetics of the antimicrobial *in vivo*, host status, and clinical trials, to identify the lowest MIC to give a good clinical outcome. Ideally, the MIC should be at the lower end of the achievable tissue concentration. However, *in vitro* resistance does not necessarily equate to treatment failure. The clinical breakpoints of topically applied antimicrobials are unknown, and values for the topical treatment of corneal infection are based on achievable and safe serum concentrations that may not be relevant for topically applied antimicrobials. Suggested ophthalmic breakpoints for bacteria and fungi are provided by regulatory authorities such as EUCAST or CSLI.[13,14] For microbial keratitis, it is difficult to establish a breakpoint without outcome data, so defining the epidemiological cut-off value (ECV or ECOFF) is an alternative. The ECOFF is not equivalent to a breakpoint but is the MIC that separates isolates into those with or without acquired or mutational resistance by defining the upper limit of the wild-type MIC distribution. Again, it does not predict clinical success but indicates whether a specific isolate carries resistance to an antimicrobial that would otherwise be active against that species.

For peer review only

AMR is an intrinsic or acquired characteristic encoded by genes that can be transferred between bacteria.[15] It is a global phenomenon that has made some systemic bacterial infections challenging to treat.[16–19] An increase in the prevalence of bacterial resistance to fluoroquinolones, beta-lactams and aminoglycosides has been reported in ophthalmic isolates.[8,18,20–24] Although *P. aeruginosa* susceptibility to either ciprofloxacin or moxifloxacin for corneal isolates is still approximately 80% worldwide,[8,23–26] several studies have highlighted the presence of high proportions of AMR bacteria in ocular infections, particularly in the US,[27] China,[28] and India.[29] A particularly sharp increase in resistance of *P. aeruginosa* to moxifloxacin occurred between 2007 and 2009 in southern India, from 19% (95% CI 5.4% to 41.9%) to 52% (95% CI 29.8% to 74.3%) (P=0.024).[30] Two separate 20-year reviews from the US also found an increase in methicillin-resistant *S. aureus* keratitis from 1993 to 2015.[23,31] Fluoroquinolone-resistance, methicillin-resistance and multidrug-resistant (MDR) bacteria, defined as acquired resistance to at least one antimicrobial agent in three or more antimicrobial classes, has also been reported in isolates from bacterial keratitis.[25,32–35] *P. aeruginosa* can show MDR,[36,37] with MDR *P. aeruginosa* emerging as a cause of bacterial keratitis in South Asia.[38,39] In South China, an increase in AMR in Gram-positive cocci was reported between 2010 to 2018, while the susceptibility of Gram-negative bacilli to fluoroquinolones and aminoglycosides was stable.[28]

Evidence for a change in AMR to the agents used to treat fungal or acanthamoeba keratitis is less certain. Susceptibility testing for antifungal agents is usually only performed in large hospitals or national reference laboratories,[40] and susceptibility testing for antiamoebic agents is rarely performed. The prevalence of acquired resistance to antifungal agents is generally lower than antibiotic resistance, but the treatment options are more limited.[41,42] Most data on the prevalence of resistance to antifungal agents is derived from testing isolates from systemic

For peer review only

infections, with relatively few studies of corneal isolates.[43–46] Methods to assess fungal resistance include broth dilution, disc dilution, antifungal impregnated gradient strips and agar screening as described by EUCAST or the CSLI. Resistance may also be intrinsic (e.g., resistance of *Candida krusei* to fluconazole) or an acquired trait following prolonged drug exposure. If resistance is uncommon (e.g., *Aspergillus fumigatus* to voriconazole), empiric treatment can be determined by accurately identifying the pathogen without routine susceptibility testing. In addition, if there is no interpretive data (breakpoint or ECOFF), the MIC alone may be of limited clinical value. For isolates from systemic infection, there is a trend toward infection with non-*albicans* species of *Candida* and an increase in antifungal resistance,[47] and acquired resistance has emerged in species such as *Candida glabrata* and *Candida auris* (rarely reported as an ocular pathogen). There has also been an increase in the resistance of *Aspergillus fumigatus* to azoles, especially in Europe,[48] linked to the use of azoles in agriculture.[49] However, polyenes (natamycin, amphotericin B) are preferred for empiric therapy of fungal keratitis rather than azoles.[50,51] Therapeutic guidelines for fungal keratitis have largely been developed from randomised controlled trials (RCTs) performed in South Asia.[50–52] Importantly, the the vast majority of isolates from these studies were filamentous fungi, although yeast contributes approximately 25% of isolates in case series from temperate regions.[53] Comparisons of antifungal resistance between regions for isolates from keratitis have not been performed.

Speciation of isolates of *Acanthamoeba* spp. or susceptibility testing against biocides is not usually performed or discussed by the EUCAST or CSLI. There is a lack of consensus on the optimum protocol for testing trophozoites or cysts against antiseptics (polyhexanide, chlorhexidine).[54] When it is done, susceptibility testing is performed on cysts because most amoebicides readily kill trophozoites. The usual test is based on preventing excystment after culture, or assessing cyst viability by staining with trypan blue, following exposure to serial

For peer review only

dilutions for one to seven days *in vitro*.[55,56] However, this protocol is primarily used for the assessment of potential amoebicidal drugs rather than for clinical management or audit of resistance. A few publications that have used these methods show a poor correlation between *in vitro* susceptibility and clinical eradication of acanthamoeba from the cornea.[57,58]

A recent systematic review and meta-analysis estimated differences in the spectrum of bacterial isolates between regions, the overall prevalence of bacterial AMR, and changes over time.[7] However, there have been no reviews of resistance in either fungal or amoebic keratitis. The review by Zhang et al. was limited to citations in English published after 2000, with an emphasis on reporting positive culture rates and the spectrum of bacterial isolates globally, with no regional data.[7] We will extend the study to all microbes, and include all publications with no limit to time or language. We will report differences in resistance between Global Burden of Disease (GBD) regions. Because ophthalmic breakpoints are unknown, we will also record the MIC data where this is available. This is because reporting the prevalence of resistance does not identify the primary measurement, which is the MIC. In addition, changes in MIC can occur before the effect of this is evident as a change in resistance values (MIC drift). Establishing the prevalence of antimicrobial resistance may focus attention on the magnitude of the problem and serve as a benchmark for future publications.

Objectives

- To report the proportion of the common pathogens isolated from microbial keratitis that are resistant to the antimicrobials commonly used as empiric therapy and report the distribution of their MICs.
- To explore how the proportion of AMR and the distribution of MICs varies by region.

For peer review only

- To explore if the proportion of AMR and the distribution of MICs in common pathogens has changed over time.

METHODS AND ANALYSIS

Protocol and registration

We report this protocol following the Preferred Reporting Items for Systematic Reviews and Meta-Analyses Protocols (PRISMA-P) guidelines.[59] The protocol has been registered in PROSPERO (registration number CRD42023331126). Any protocol amendments will be documented, including the date of amendment and rationale for the change in the final review report.

Types of studies

We will include published prospective and retrospective cross-sectional studies that have identified the genus and species of isolates from corneal cultures and recorded the susceptibility or MIC of each isolate to eye-appropriate topical antimicrobials. We define eye-appropriate antimicrobials as agents that are either commercially available or specifically compounded for topical ophthalmic use. We will also consider the baseline findings (pre-randomisation) of people recruited in clinical trials for treatment or cohort studies. We will only include peer-reviewed articles published in any language. Articles in a language other than English will be screened with an internet-based translation service (e.g., Google Translate). If they appear relevant a professional translation will be obtained. We will not include grey literature or conference abstracts. We will consider publications from all years and geographical regions.

Types of participants, eligibility, and setting

Inclusion criteria will be studies that report the investigation results for suspected microbial keratitis, including bacterial, fungal and acanthamoeba keratitis. We will exclude studies that only report on viral isolates or *in vitro* resistance to antivirals. There will be no age restrictions.

Settings will include primary care as well as secondary and tertiary referral centres. Studies must report the results of an investigation, including the identification of the organisms grown in culture (genus and species) and their anticipated susceptibility or resistance to antimicrobials, with the measured MIC if this is available. The genus and species will be recorded. The method of assessment (disc diffusion, Etest, tube dilution) will be recorded. The regulatory body that provided the assessment thresholds or breakpoints will be recorded, such as EUCAST, CSLI, ChiCAST. We will include case series, but not individual case reports, as we require a representative sample of isolates from a particular setting with information on the proportion with AMR. We will exclude studies where participants are exclusively patients in specific settings (e.g., intensive care units), or patients with specific syndromes (e.g., Stevens Johnson syndrome) or other disease groups (non-healing ulcers, corneal graft for treatment failure). We will include studies irrespective of pre-treatment of patients. We will group the results as bacteria, fungi or acanthamoeba, and we will pool data from studies within regions. The absolute number of isolates and the proportion of the total isolates will be recorded. Differences between the method of identification (MALDI-TOF vs biochemical assay) for bacteria is not thought to be significant. Results from PCR will not be included as these do not routinely measure AMR. We will not analyse the response to therapy.

For peer review only

Types of outcome measures

- The proportion of the common pathogens isolated from suspected microbial keratitis that are resistant to the antimicrobials commonly used as empiric therapy. Results will be recorded as sensitive (S), sensitive at an increased dosage (I), or resistant (R). For the analysis the S and I groups will be amalgamated.
- The MIC values (mg/L) for the antimicrobials commonly used as empiric therapy for suspected microbial keratitis. Results will be reported as the mode and range.

For peer review only - http://bmjopen.bmj.com/site/about/guidelines.xhtml

Search strategies

Electronic searches

We will conduct an electronic bibliographic search in MEDLINE, EMBASE, Web of Science, and the Cochrane Library. An expert information specialist developed the search strategies (Supplementary Figure 1). Relevant publications will be retrieved manually if electronic access is not available.

Searching for other resources

We will identify additional studies by searching the reference lists of relevant publications identified through electronic searches and prior review articles on this topic. We will consult trial registries such as WHO ICTRP and ClinicalTrials.gov to identify studies indexed in the databases.

Selection of studies

Two review authors will independently screen search results based on title and abstract and will remove reports that do not fall into the scope of this review. We will resolve disagreements by discussion and consultation with another author as needed. We will acquire the full text of all publications appearing to meet the criteria for inclusion in this review. Two review authors will independently screen the full-text reports using the inclusion criteria listed above. They will discuss any disagreements, and a third review author will arbitrate if they cannot resolve them. Screening of search results will be conducted using Covidence systematic review software (Veritas Health Innovation, Melbourne, Australia; available at www.covidence.org).

Data extraction and management

Data extraction will be done in Covidence using a customised data extraction template which will be pre-piloted on 5 studies (Table 1). Two review authors will independently extract the following data from each study: study design, participant characteristics, study population and size, study setting, study dates, diagnostic and ascertainment methods, details of pathogen and

review only

antimicrobials, number of isolates, the proportion of isolates reported resistant (R) for each antimicrobial, and the MIC values of these antimicrobials. We will document the method used for speciation of the organism, how the MIC was determined, and the reference guidelines for determining breakpoint thresholds. Disagreements will be resolved by discussion and consultation with another author as needed; a third review author will arbitrate if they cannot resolve them.

AMR will be recorded separately for bacteria, fungi and acanthamoeba. Within each phylum, the AMR will be reported for each class of antimicrobial against each genus of organism.

- For bacterial isolates, the genus and species of all isolates will be recorded, with a secondary grouping of pathogens into classes. The groups will be: *Enterobacterales, Pseudomonas* spp., *Staphylococcus* spp., *methicillin-resistant S. aureus* (MRSA), *coagulase-negative staphylococci (CNS)*, *Streptococcus pneumoniae,* viridans group streptococci *(VGS)*, *beta-haemolytic streptococci*, *Haemophilus* spp., *Neisseria* spp*.* The antimicrobial groups will be quinolones (second and subsequent generations), beta-lactams (early and late cephalosporins), aminoglycosides, chloramphenicol, glycopeptides and antiseptics.
- For fungal isolates, the genus and species will be recorded, with a secondary grouping of the pathogens into classes. The groups will be yeast and filamentous fungi. The antifungal groups will be the polyenes (natamycin, amphotericin), azoles (miconazole, econazole, voriconazole), echinocandins (micafungin) and antiseptics.
- For amoeba isolates the genus will be recorded and the species if this has been reported. The anti-amoebic treatments include polyhexanide, chlorhexidine, voriconazole and neomycin.

For peer review only

Table 1. Data extraction domains for included studies

	Domain/subdomain	Description
1	***Document characteristics***	
	Title, authors, publication year	Title, authors, and year of publication
	Full citation and web link	Citation of publication and PMID
2	***Study characteristics***	
	Design	Prospective study, retrospective study, RCT, etc.
	Setting	Primary care, hospital eye service, tertiary referral centre, number of participating centres
	Location	City and country of data collection, GBD region
	Data collection	Years when samples collected
	Population	Eligibility criteria, age range, specific risk group (LVC, ICU, SJS)
	Sample size	Number of participants in the study. Eyes included.
	Study objectives	What was the study research question?
3	***Characteristics***	
	Inclusion criteria	Presumed microbial keratitis, size and position of ulcer
	Exclusion criteria	Case report, selected disease group, e.g., corneal perforation
	Definition of significant isolate	Growth on one or more media, supportive investigation (histology, PCR, IVCM)
	Microbial isolate	Bacteria, fungus, acanthamoeba

For peer review only

	Method of identification	e.g., MALDI-TOF, phenotypic and biochemical, molecular
	Determination of MIC	Tube dilution, Etest, disc diffusion, agar slope, excystment.
	Reference body for breakpoint	EUCAST, CSLI, ChiCAST
	Bacterial groups	Enterobacteriales, Pseudomonas spp, Staphylococcus spp, MRSA, Staphylococcus (CNS), Streptococcus pneumonia, Streptococcus (VTS), beta-haemolytic Streptococcus, Haemophilus spp, Neisseria spp.
	Sensitivity and MIC of bacteria to antimicrobials (6 categories)	Quinolones, beta-lactam, aminoglycoside, glycopeptide, chloramphenicol, antiseptics
	Fungal groups	Yeast, filamentary fungus
	Sensitivity and MIC of fungi to antifungals (4 categories)	Polyenes, azoles, echinocandins, antiseptics
	Amoeba isolates	Acanthamoeba with speciation if available
	Sensitivity and MIC of amoeba to amebicides (4 categories)	Polyhexanide, chlorhexidine, voriconazole, neomycin

MIC: minimum inhibitory concentration. EUCAST: European Committee on Antimicrobial Susceptibility Testing. CSLI: Clinical & Laboratory Standards Institute. MRSA: methicillin-resistant Staphylococcus aureus. VTS: viridans-type streptococci. MALDI-TOF: matrix-assisted laser desorption/ionization time of flight. LVC: laser vision correction. ICU: intensive care unit. SJS: Stevens Johnson syndrome. PCR: polymerase chain reaction. IVCM: in vivo confocal microscopy. GBD: Global Burden of Disease. RCT: randomised control trial.

Assessment of risk of bias

Two reviewers will independently assess the risk of bias in each included study using the risk of bias tool for prevalence studies developed by Hoy et al.[60] This tool covers four domains - selection bias, non-response bias, measurement bias and bias related to analysis. For each of

For peer review only

For peer review only - http://bmjopen.bmj.com/site/about/guidelines.xhtml

the 10 items included in the assessment, two reviewers will record the rationale for their judgements. The Risk of Bias (Supplementary Figure 2) shows the items included in the tool and specific considerations for this review. This risk of bias tool will be piloted on 5 studies and instructions for reviewers amended in the light of that pilot. Agreement between reviewers will be assessed and any disagreements resolved by discussion. For each study, a judgement of the overall risk of bias (low, moderate, or high) will be made.

Dealing with missing data

If we do not find all the necessary information in a published study, for articles published in 2010 or later, we will email the corresponding author to solicit further information. If we cannot obtain the necessary information, we will document in the review that we attempted to contact the study authors. We will report and discuss the possible effect of missing data on each study and the overall review and meta-analysis. We will consider the susceptibility of our meta-analysis to the impact of missing data. We will analyse the data that is available rather than imputing missing data.

Unit of analysis issues

We will document how each study handled eyes - whether one eye or both eyes were included and analysed in the study. We will also document how the study handled multiple isolates per person or eye. Where multiple isolates are identified we will consider each isolate separately and analyse as reported. Where possible, we will extract proportions/confidence intervals adjusted for clustered data or consider adjusting using methods outlined in the Cochrane Handbook.[61]

Data synthesis and meta-analysis approach

We will perform meta-analyses using a random-effects model in Stata V.17 (StataCorp).[62] We will summarise and report the pooled proportion of resistance, its 95% CI, and between-study

For peer review only

heterogeneity (I^2). We will conduct meta-analyses separately for each class of antimicrobial (Table 1): fluoroquinolones (second and fourth generation separately), beta-lactam antibiotics (e.g., cefuroxime), aminoglycosides (e.g., gentamicin, tobramycin), glycopeptide antibiotics (e.g., vancomycin) for bacterial; natamycin, voriconazole, amphotericin, (and others) for fungi (filamentous fungi, yeasts); polyhexanide (PHMB) and voriconazole for *Acanthamoeba* spp. If a study reports susceptibility for more than one antimicrobial in a class against the same organism(s), our strategy for the primary analysis will be to select the results for the most frequently used antimicrobial of that class from the whole data set. We will then perform a sensitivity analysis substituting results for the less commonly used antibiotic(s) in that class to check that the results do not change substantially. We will consider amalgamating closely related groups of organisms (Table 1) that have similar mechanisms of AMR.

We anticipate heterogeneity in reporting of MIC values and therefore we plan to report these data narratively in structured tables and figures. If comparable figures on MIC in mg/L are available from different studies, we will consider a meta-analysis as specified above.

Meta regression

If there are sufficient studies for analysis, we will include the following covariates:

- Class of microbe: We intend to present data for the eight most frequent Gram-positive (*S. aureus*, *Streptococcus* spp. etc) and Gram-negative (*P. aeruginosa*, etc) isolates.
- Region: we will group studies according to the global super-region as defined by the Global Burden of Disease Study.[63]
- Decade: we will group studies according to the decade in which they were conducted: Before 2000, 2000-2009, 2010-2019, 2020 - 2022.
- Pre-treatment (none, some, all).

For peer review only

- Sex (M:F)
- Age (children:adults >16 years)
- Study size. We will assess small study effects, one of which may be publication bias, by preparing a funnel plot, which is a scatter plot of effect size versus precision (standard error).[64]

Assessment of heterogeneity

We will assess clinical heterogeneity by comparing key participant characteristics at the study level (e.g., age, sex, ocular diagnosis). Methodological heterogeneity will also be considered, including comparing the risk of bias of included studies. We will assess statistical heterogeneity by inspecting forest plots and through the I^2 statistic. If high levels of inconsistency are detected, and there are enough studies, we will explore likely sources of this heterogeneity (see section on meta-regression).

Sensitivity analysis

We will conduct a sensitivity analysis of the primary analysis in which studies are excluded if they are judged to be at high risk of bias.

Assessing the certainty of the evidence

We will assess and report the overall certainty of evidence from our analyses and critical appraisal using the modified Grades of Recommendation, Assessment, Development and Evaluation tool.[65] We will consider evidence from cross-sectional studies to be high certainty and will downgrade for risk of bias, imprecision, inconsistency, indirectness and publication bias. The GRADE assessment will be done by consensus discussion.

Patient and public involvement

None.

For peer review only

ETHICS AND DISSEMINATION

Ethics approval is not required as this is a protocol for a systematic review of published data. Findings will be published in an open-access peer-reviewed journal and presented at national and international meetings. We anticipate that the findings will be of considerable interest to those involved in eye health provision, as well as the general medical, public health, development, and governmental sectors.

*** ***

Contributors

ST conceived the original idea. ST and JE wrote the first draft of this protocol, and MB, TN, DM, SK, IG, AL, and NS made further suggestions. ST, JE, DM, NS, TN, AL, SK, and MB participated in the study's design and the inclusion and exclusion criteria setting. IG, JE, and ST developed the search strategy. ST, JE, MB, SK, and DM will supervise the data extraction and the overall work. ST and JE are the guarantors. All authors read the final draft and approved the publication of the protocol.

Funding

MJB, AL and DM and supported by Wellcome Trust (207472/Z17/Z). The funder had no role in the development of the review.

Competing interests

None declared.

Provenance and peer review

Not commissioned, externally peer reviewed.

For peer review only

References

1 Brown L, Leck AK, Gichangi M, *et al.* The global incidence and diagnosis of fungal keratitis. *Lancet Infect Dis* 2021;**21**:e49–57.

2 Song X, Xie L, Tan X, *et al.* A multi-center, cross-sectional study on the burden of infectious keratitis in China. *PLoS One* 2014;**9**:e113843.

3 Stapleton F. The epidemiology of infectious keratitis. *Ocul Surf* Published Online First: 19 August 2021. doi:10.1016/j.jtos.2021.08.007

4 Erie JC, Nevitt MP, Hodge DO, *et al.* Incidence of ulcerative keratitis in a defined population from 1950 through 1988. *Arch Ophthalmol* 1993;**111**:1665–71.

5 Lin A, Rhee MK, Akpek EK, *et al.* Bacterial Keratitis Preferred Practice Pattern®. *Ophthalmology* 2019;**126**:P1–55.

6 Ung L, Wang Y, Vangel M, *et al.* Validation of a Comprehensive Clinical Algorithm for the Assessment and Treatment of Microbial Keratitis. *Am J Ophthalmol* 2020;**214**:97–109.

7 Zhang Z, Cao K, Liu J, *et al.* Pathogens and Antibiotic Susceptibilities of Global Bacterial Keratitis: A Meta-Analysis. *Antibiotics (Basel)* 2022;**11**. doi:10.3390/antibiotics11020238

8 Lalitha P, Manoharan G, Karpagam R, *et al.* Trends in antibiotic resistance in bacterial keratitis isolates from South India. *Br J Ophthalmol* 2017;**101**:108–13.

9 Srinivasan M, Mascarenhas J, Rajaraman R, *et al.* Corticosteroids for bacterial keratitis: the Steroids for Corneal Ulcers Trial (SCUT). *Arch Ophthalmol* 2012;**130**:143–50.

10 Bharathi MJ, Ramakrishnan R, Vasu S, *et al.* Aetiological diagnosis of microbial keratitis in South India - a study of 1618 cases. *Indian J Med Microbiol* 2002;**20**:19–24.

For peer review only

11 Shah A, Sachdev A, Coggon D, *et al.* Geographic variations in microbial keratitis: an analysis of the peer-reviewed literature. *Br J Ophthalmol* 2011;**95**:762–7.

12 Arunga S, Kintoki GM, Mwesigye J, *et al.* Epidemiology of Microbial Keratitis in Uganda: A Cohort Study. *Ophthalmic Epidemiol* 2020;**27**:121–31.

13 Clinical & Laboratory Standards Institute: CLSI guidelines. Clinical & Laboratory Standards Institute. https://clsi.org/ (accessed 18 Mar 2022).

14 ESCMID-European Society of Clinical Microbiology, Diseases I. EUCAST: EUCAST. https://eucast.org/ (accessed 18 Mar 2022).

15 Blair JMA, Webber MA, Baylay AJ, *et al.* Molecular mechanisms of antibiotic resistance. *Nat Rev Microbiol* 2015;**13**:42–51.

16 Leibovitch I, Lai TF, Senarath L, *et al.* Infectious keratitis in South Australia: emerging resistance to cephazolin. *Eur J Ophthalmol* 2005;**15**:23–6.

17 Shalchi Z, Gurbaxani A, Baker M, *et al.* Antibiotic resistance in microbial keratitis: ten-year experience of corneal scrapes in the United Kingdom. *Ophthalmology* 2011;**118**:2161–5.

18 Alexandrakis G, Alfonso EC, Miller D. Shifting trends in bacterial keratitis in south Florida and emerging resistance to fluoroquinolones. *Ophthalmology* 2000;**107**:1497–502.

19 Littmann J, Viens AM. The Ethical Significance of Antimicrobial Resistance. *Public Health Ethics* 2015;**8**:209–24.

20 Ray KJ, Prajna L, Srinivasan M, *et al.* Fluoroquinolone treatment and susceptibility of isolates from bacterial keratitis. *JAMA Ophthalmol* 2013;**131**:310–3.

21 Goldstein MH, Kowalski RP, Gordon YJ. Emerging fluoroquinolone resistance in bacterial

For peer review only

keratitis: a 5-year review. *Ophthalmology* 1999;**106**:1313–8.

22 Garg P, Sharma S, Rao GN. Ciprofloxacin-resistant Pseudomonas keratitis. *Ophthalmology* 1999;**106**:1319–23.

23 Peng MY, Cevallos V, McLeod SD, *et al.* Bacterial Keratitis: Isolated Organisms and Antibiotic Resistance Patterns in San Francisco. *Cornea* 2018;**37**:84–7.

24 Ni N, Nam EM, Hammersmith KM, *et al.* Seasonal, geographic, and antimicrobial resistance patterns in microbial keratitis: 4-year experience in eastern Pennsylvania. *Cornea* 2015;**34**:296–302.

25 Kaliamurthy J, Kalavathy CM, Parmar P, *et al.* Spectrum of bacterial keratitis at a tertiary eye care centre in India. *Biomed Res Int* 2013;**2013**:181564.

26 Lichtinger A, Yeung SN, Kim P, *et al.* Shifting trends in bacterial keratitis in Toronto: an 11-year review. *Ophthalmology* 2012;**119**:1785–90.

27 Asbell PA, Sanfilippo CM, Sahm DF, *et al.* Trends in Antibiotic Resistance Among Ocular Microorganisms in the United States From 2009 to 2018. *JAMA Ophthalmol* 2020;**138**:439–50.

28 Lin L, Duan F, Yang Y, *et al.* Nine-year analysis of isolated pathogens and antibiotic susceptibilities of microbial keratitis from a large referral eye center in southern China. *Infect Drug Resist* 2019;**12**:1295–302.

29 Singh M, Gour A, Gandhi A, *et al.* Demographic details, risk factors, microbiological profile, and clinical outcomes of pediatric infectious keratitis cases in North India. *Indian J Ophthalmol* 2020;**68**:434–40.

30 Oldenburg CE, Lalitha P, Srinivasan M, *et al.* Emerging moxifloxacin resistance in

For peer review only

Pseudomonas aeruginosa keratitis isolates in South India. *Ophthalmic Epidemiol* 2013;**20**:155–8.

31 Chang VS, Dhaliwal DK, Raju L, *et al.* Antibiotic Resistance in the Treatment of Staphylococcus aureus Keratitis: a 20-Year Review. *Cornea* 2015;**34**:698–703.

32 Cariello AJ, Passos RM, Yu MCZ, *et al.* Microbial keratitis at a referral center in Brazil. *Int Ophthalmol* 2011;**31**:197–204.

33 Acharya M, Farooqui J, Gaba T, *et al.* Delhi infectious keratitis study: Update on clinico-Microbiological profile and outcomes of infectious keratitis. Journal of Current Ophthalmology. 2020;**32**:249. doi:10.4103/joco.joco_113_20

34 Hernandez-Camarena JC, Graue-Hernandez EO, Ortiz-Casas M, *et al.* Trends in Microbiological and Antibiotic Sensitivity Patterns in Infectious Keratitis. Cornea. 2015;**34**:778–85. doi:10.1097/ico.0000000000000428

35 Vola ME, Moriyama AS, Lisboa R, *et al.* Prevalence and antibiotic susceptibility of methicillin-resistant Staphylococcus aureus in ocular infections. *Arq Bras Oftalmol* 2013;**76**:350–3.

36 Magiorakos A-P, Srinivasan A, Carey RB, *et al.* Multidrug-resistant, extensively drug-resistant and pandrug-resistant bacteria: an international expert proposal for interim standard definitions for acquired resistance. *Clin Microbiol Infect* 2012;**18**:268–81.

37 Tacconelli E, Carrara E, Savoldi A, *et al.* Discovery, research, and development of new antibiotics: the WHO priority list of antibiotic-resistant bacteria and tuberculosis. *Lancet Infect Dis* 2018;**18**:318–27.

38 Fernandes M, Vira D, Medikonda R, *et al.* Extensively and pan-drug resistant

For peer review only

Pseudomonas aeruginosa keratitis: clinical features, risk factors, and outcome. *Graefes Arch Clin Exp Ophthalmol* 2016;**254**:315–22.

39 Vazirani J, Wurity S, Ali MH. Multidrug-Resistant Pseudomonas aeruginosa Keratitis: Risk Factors, Clinical Characteristics, and Outcomes. *Ophthalmology* 2015;**122**:2110–4.

40 Berkow EL, Lockhart SR, Ostrosky-Zeichner L. Antifungal Susceptibility Testing: Current Approaches. *Clin Microbiol Rev* 2020;**33**. doi:10.1128/CMR.00069-19

41 Odds FC, Brown AJP, Gow NAR. Antifungal agents: mechanisms of action. *Trends Microbiol* 2003;**11**:272–9.

42 Perlin DS, Rautemaa-Richardson R, Alastruey-Izquierdo A. The global problem of antifungal resistance: prevalence, mechanisms, and management. *Lancet Infect Dis* 2017;**17**:e383–92.

43 Oechsler RA, Feilmeier MR, Miller D, *et al.* Fusarium keratitis: genotyping, in vitro susceptibility and clinical outcomes. *Cornea* 2013;**32**:667–73.

44 Lalitha P, Sun CQ, Prajna NV, *et al.* In vitro susceptibility of filamentous fungal isolates from a corneal ulcer clinical trial. *Am J Ophthalmol* 2014;**157**:318–26.

45 Mukherjee PK, Chandra J, Yu C, *et al.* Characterization of fusarium keratitis outbreak isolates: contribution of biofilms to antimicrobial resistance and pathogenesis. *Invest Ophthalmol Vis Sci* 2012;**53**:4450–7.

46 Kimura K, Inoue Y, Asari S, *et al.* Multicenter prospective observational study of fungal keratitis in Japan: analyses of in vitro susceptibility tests for combinations of drugs. *Jpn J Ophthalmol* 2022;**66**:240–53.

47 Gonçalves SS, Souza ACR, Chowdhary A, *et al.* Epidemiology and molecular mechanisms

For peer review only

of antifungal resistance in Candida and Aspergillus. *Mycoses* 2016;**59**:198–219.

48 Resendiz Sharpe A, Lagrou K, Meis JF, *et al.* Triazole resistance surveillance in Aspergillus fumigatus. *Med Mycol* 2018;**56**:83–92.

49 Chowdhary A, Kathuria S, Xu J, *et al.* Correction: Emergence of Azole-Resistant Aspergillus fumigatus Strains due to Agricultural Azole Use Creates an Increasing Threat to Human Health. PLoS Pathogens. 2013;**9**. doi:10.1371/annotation/4ffcf1da-b180-4149-834c-9c723c5dbf9b

50 Hoffman JJ, Yadav R, Sanyam SD, *et al.* Topical Chlorhexidine 0.2% versus Topical Natamycin 5% for the Treatment of Fungal Keratitis in Nepal: A Randomized Controlled Noninferiority Trial. *Ophthalmology* Published Online First: 9 December 2021. doi:10.1016/j.ophtha.2021.12.004

51 Prajna NV, Krishnan T, Mascarenhas J, *et al.* The mycotic ulcer treatment trial: a randomized trial comparing natamycin vs voriconazole. *JAMA Ophthalmol* 2013;**131**:422–9.

52 Prajna NV, Krishnan T, Rajaraman R, *et al.* Effect of Oral Voriconazole on Fungal Keratitis in the Mycotic Ulcer Treatment Trial II (MUTT II): A Randomized Clinical Trial. *JAMA Ophthalmol* 2016;**134**:1365–72.

53 Ong HS, Fung SSM, Macleod D, *et al.* Altered Patterns of Fungal Keratitis at a London Ophthalmic Referral Hospital: An Eight-Year Retrospective Observational Study. *Am J Ophthalmol* 2016;**168**:227–36.

54 Shing B, Balen M, McKerrow JH, *et al.* Acanthamoeba Keratitis: an update on amebicidal and cysticidal drug screening methodologies and potential treatment with azole drugs. *Expert Rev Anti Infect Ther* 2021;**19**:1427–41.

For peer review only

For peer review only - http://bmjopen.bmj.com/site/about/guidelines.xhtml

55 Narasimhan S, Madhavan HN, K LT. Development and application of an in vitro susceptibility test for Acanthamoeba species isolated from keratitis to polyhexamethylene biguanide and chlorhexidine. *Cornea* 2002;**21**:203–5.

56 Turner NA, Russell AD, Furr JR, *et al.* Emergence of resistance to biocides during differentiation of Acanthamoeba castellanii. *J Antimicrob Chemother* 2000;**46**:27–34.

57 Elder MJ, Kilvington S, Dart JK. A clinicopathologic study of in vitro sensitivity testing and Acanthamoeba keratitis. *Invest Ophthalmol Vis Sci* 1994;**35**:1059–64.

58 Pérez-Santonja JJ, Kilvington S, Hughes R, *et al.* Persistently culture positive acanthamoeba keratitis: in vivo resistance and in vitro sensitivity. *Ophthalmology* 2003;**110**:1593–600.

59 Moher D, Shamseer L, Clarke M, *et al.* Preferred reporting items for systematic review and meta-analysis protocols (PRISMA-P) 2015 statement. *Syst Rev* 2015;**4**:1.

60 Hoy D, Brooks P, Woolf A, *et al.* Assessing risk of bias in prevalence studies: modification of an existing tool and evidence of interrater agreement. *J Clin Epidemiol* 2012;**65**:934–9.

61 Higgins JPT, Eldridge S, Li T. Including variants on randomized trials. Cochrane Handbook for Systematic Reviews of Interventions. 2019;:569–93. doi:10.1002/9781119536604.ch23

62 Nyaga VN, Arbyn M, Aerts M. Metaprop: a Stata command to perform meta-analysis of binomial data. *Arch Public Health* 2014;**72**:39.

63 GBD regions and super regions. The International Agency for the Prevention of Blindness. 2020.https://www.iapb.org:8443/learn/vision-atlas/about/definitions-and-regions/ (accessed 23 Feb 2022).

64 Egger M, Davey Smith G, Schneider M, *et al.* Bias in meta-analysis detected by a simple,

For peer review only

graphical test. *BMJ* 1997;**315**:629–34.

65 Iorio A, Spencer FA, Falavigna M, *et al.* Use of GRADE for assessment of evidence about prognosis: rating confidence in estimates of event rates in broad categories of patients. *BMJ* 2015;**350**:h870.

For peer review only

Supplementary figure 1
Search strategy

MEDLINE
1. keratitis/ or acanthamoeba keratitis/
2. keratitis.tw.
3. (fung$ adj3 infect$ adj3 eye$).tw.
4. (fung$ adj3 infect$ adj3 ocular).tw.
5. (bacteria$ adj3 infect$ adj3 eye$).tw.
6. (bacteria$ adj3 infect$ adj3 ocular).tw.
7. or/1-6
8. exp Drug Resistance, Microbial/
9. ((antimicrobial or antifungal or increase$) adj2 resistan$).tw.
10. "AMR".tw.
11. Microbial Sensitivity Tests/
12. (drug adj2 (resistan$ or suscept$)).tw.
13. (minimum adj1 inhibit$ adj1 concentration$).tw.
14. "MIC".tw.
15. breakpoint.tw.
16. or/8-15
17. prevalence/
18. prevalence.tw.
19. case-control studies/ or retrospective studies/ or cohort studies/ or follow-up studies/ or longitudinal studies/ or prospective studies/ or cross-sectional studies/ or observational study/
20. (case adj1 control$ adj3 (stud$ or trial$)).tw.
21. (cross adj1 sectional$ adj3 (stud$ or trial$)).tw.
22. (follow adj1 up adj3 (stud$ or trial$)).tw.
23. ((prospective or retrospective or cohort or longitudinal or observational) adj3 (stud$ or trial$)).tw.
24. clinical trial/ or control groups/ or double-blind method/ or meta-analysis as topic/ or network meta-analysis/ or random allocation/ or single-blind method/
25. randomized controlled trial/ or controlled clinical trials as topic/ or randomized controlled trials as topic/ or comparative study/ or evaluation studies/ or meta- analysis/ or "systematic review"/
26. random$.tw.
27. or/17-26
28. 7 and 16 and 27
29 (animal or animals or mouse or mice or rat or rats or rabbit$ or dog or dogs or canine or cat or cats or pig or pigs or horse or horses or veterinary).tw.
30 28 not 29

Embase
1. keratitis/ or bacterial keratitis/ or virus keratitis/ or Acanthamoeba keratitis/ or microbial keratitis/ or suppurative keratitis/ or amebic keratitis/
2. keratitis.tw.
3. (fung$ adj3 infect$ adj3 eye$).tw.
4. (fung$ adj3 infect$ adj3 ocular).tw.
5. (bacteria$ adj3 infect$ adj3 eye$).tw.
6. (bacteria$ adj3 infect$ adj3 ocular).tw.
7. or/1-6
8. antibiotic resistance/ or antibiotic sensitivity/ or drug resistance/

For peer review only

9. aminoglycoside resistance/ or beta-lactam resistance/ or chloramphenicol resistance/ or fluoroquinolone resistance/
10. ((antimicrobial or antifungal or increase$) adj2 resistan$).tw.
11. "AMR".tw.
12. microbial sensitivity test/
13. (drug adj2 (resistan$ or suscept$)).tw.
14. minimum inhibitory concentration/
15. (minimum adj1 inhibit$ adj1 concentration$).tw.
16. "MIC".tw.
17. breakpoint.tw.
18. or/8-17
19. prevalence/
20. prevalence.tw.
21. controlled clinical trial/
22. case control study/
23. cohort analysis/
24. follow up/
25. longitudinal study/
26. observational study/
27. prospective study/
28. retrospective study/
29. cross-sectional study/
30. control group/
31. "meta analysis (topic)"/
32. network meta-analysis/
33. randomization/
34. single blind procedure/
35. double blind procedure/
36. control group/
37. controlled clinical trial/
38. clinical study/
39. clinical trial/
40. "clinical trial (topic)"/
41. "controlled clinical trial (topic)"/
42. "randomized controlled trial (topic)"/
43. comparative study/
44. evaluation study/
45. randomized controlled trial/
46. meta analysis/
47. "systematic review"/
48. "systematic review (topic)"/
49. (case adj1 control$ adj3 (stud$ or trial$)).tw.
50. (cross adj1 sectional$ adj3 (stud$ or trial$)).tw.
51. (follow adj1 up adj3 (stud$ or trial$)).tw.
52. ((prospective or retrospective or cohort or longitudinal or observational) adj3 (stud$ or trial$)).tw.
53. random$.tw.
54. or/19-53
55. 7 and 18 and 54
56. (animal or animals or mouse or mice or rat or rats or rabbit$ or dog or dogs or canine or cat or cats or pig or pigs or horse or horses or veterinary).tw.

For peer review only

57. 55 not 56

Cochrane Library

#1 MeSH descriptor: [Keratitis] this term only
#2 MeSH descriptor: [Acanthamoeba Keratitis] this term only
#3 keratitis
#4 fung* NEAR/3 infect* NEAR/3 eye
#5 fung* NEAR/3 infect* NEAR/3 ocular
#6 bacteria* NEAR/3 infect* NEAR/3 eye*
#7 bacteria* NEAR/3 infect* NEAR/3 ocular
#8 #1 or #2 or #3 or #4 or #5 or #6 or #7
#9 MeSH descriptor: [Drug Resistance, Microbial] explode all trees
#10 (antimicrobial or antifungal or increase*) NEAR/2 resistan*
#11 AMR
#12 MeSH descriptor: [Microbial Sensitivity Tests] this term only
#13 drug NEAR/2 (resistan* or suscept*)
#14 minimum NEXT inhibit* NEXT concentration*
#15 MIC
#16 breakpoint
#17 #9 or #10 or #11 or #12 or #13 or #14 or #15 or #16
#18 #8 and #17

Web of Science

#19 not #20
TS= (animal OR animals OR mouse OR mice OR rabbit* OR dog OR dogs OR canine OR horse OR horses OR veterinary)
#6 AND #11 AND #18
#17 OR #16 OR #15 OR #14 OR #13 OR #12
TS= (clinical OR comparative NEAR/3 (stud* OR trial*))
TS= (random* OR meta NEAR/1 analysis OR systematic NEAR/1 review)
TS= (follow NEAR/2 up NEAR/3 (stud* OR trial*))
TS= (cross NEAR/2 sectional* NEAR/3 (stud* OR trial*))
TS= (case NEAR/2 control* NEAR/3 (stud* OR trial*))
TS= (prevalence OR retrospective OR prospective OR cohort OR observational OR longitudinal)
#7 OR #8 OR #9 OR #10
TS= (drug NEAR/2 (resistan* or suscept*))
TS= ((antimicrobial OR antifungal OR increase*) NEAR/2 resistan*)
TS=(Microbial Sensitivity Tests)
TS=(Microbial Drug Resistance)
#1 OR #2 OR #3 OR #4 OR #5
TS= keratitis
TS= (fung* NEAR/3 infect* NEAR/3 eye*)
TS= (fung* NEAR/3 infect* NEAR/3 ocular)
TS= (bacteria* NEAR/3 infect* NEAR/3 eye*)
TS= (bacteria* NEAR/3 infect* NEAR/3 ocular)

Clinicaltrials.gov

(antimicrobial OR antifungal OR antibiotic OR drug) AND resistance | Keratitis

WHO ICTRP

For peer review only

Keratitis AND antimicrobial AND resistance OR Keratitis AND antifungal AND resistance OR Keratitis AND antibiotic AND resistance OR Keratitis AND drug AND resistance

For peer review only

Supplementary Figure 2: Risk of Bias Assessment

	**Risk of bias item**	**Criteria for answers**
1	Was the study's target population a close representation of the national population in relation to relevant variables?	Yes (LOW RISK)<br>The study's target population was a close representation of the national or regional population *e.g., population-based study or sampling of cases of microbial keratitis from representative settings.*<br>No (HIGH RISK)<br>The study's target population was clearly not representative of the national population. *e.g., sampling of cases attending tertiary referral center or cases selected from a population that had failed to improve with prior therapy with antimicrobials, selection of larger ulcers, exclusions of small peripheral ulcers or of impending or actual corneal perforation.*
2	Was the sampling frame a true or close representation of the target population?	Yes (LOW RISK): The sampling frame was a true or close representation of the target population, *e.g., an acceptable and standardized definition of microbial keratitis was used, including definitions of pathogens, standardized criteria for definition and selection of isolate type, and definition of significant growth.*<br><br>No (HIGH RISK): The sampling frame was NOT a true or close representation of the target population, e.g., *potential 'non-pathogens' were excluded. Unclear the basis for the clinical diagnosis. Definitions of pathogens and isolates are not standardized.*
3	Was some form of random selection used to select the sample, OR was a census undertaken?	Yes (LOW RISK): A census was undertaken, OR, some form of random selection was used to select the sample (e.g. simple random sampling, stratified random sampling, cluster sampling, systematic sampling), e.g. *random sample of a consecutive series of patients, or inclusion of a consecutive series of patients*<br><br>No (HIGH RISK): A census was NOT undertaken, AND some form of random selection was NOT used to select the sample *eg unclear how cases selected or unclear if consecutive or not*
4	Was the likelihood of non-response bias minimal?	Yes (LOW RISK): The response rate for the study was ≥75%, OR, an analysis was performed that showed no

For peer review only

		significant difference in relevant demographic characteristics between responders and nonresponders<br><br>No (HIGH RISK): The response rate was <75%, and if any analysis comparing responders and non-responders was done, it showed a significant difference in relevant demographic characteristics between responders and non-responders.
5	Were data collected directly from the subjects (as opposed to a proxy)?	Yes (LOW RISK): All data were collected directly from the subjects. *It is likely that data will be collected directly from all participants so this item may not be relevant and will be marked low risk of bias for all studies.*<br><br>No (HIGH RISK): In some instances, data were collected from a proxy. *Difficult to envisage a study where this would be the case.*
6	Was an acceptable and standardized case definition for AMR used in the study?	Yes (LOW RISK): An acceptable case definition was used, e.g., *definition of AMR clearly described and standardized (between geographical locations and over time if applicable)*<br><br>No (HIGH RISK): An acceptable case definition was NOT used. *e.g., unclear definition of AMR and/or definition not consistently applied in different groups of patients or over time.*
7	Was the study instrument that measured the parameter of interest shown to have validity and reliability?	Yes (LOW RISK): The study instrument had been shown to have reliability and validity (if this was necessary), e.g., test-retest, piloting, validation in a previous study, etc., *e.g., method of measuring AMR was valid and reliable i.e., valid laboratory systems for the identification of isolates and measurement of AMR or MIC.*<br><br>No (HIGH RISK): The study instrument had NOT been shown to have reliability or validity (if this was necessary), *e.g., method of measuring AMR was not clearly described or not validated.*
8	Was the same mode of data collection used for all subjects?	Yes (LOW RISK): The same mode of data collection was used for all subjects, e.g., *collection methodologies for the identified cases and measurement of resistance or MIC were adequately described and consistent during the course of the study.*

For peer review only - http://bmjopen.bmj.com/site/about/guidelines.xhtml

		No (HIGH RISK): The same mode of data collection was NOT used for all subjects, e.g., *collection methodologies for the identified and measurement of resistance or MIC not clearly described and/or inconsistent during the course of the study.*
9	Was the length of the shortest prevalence period for the parameter of interest appropriate?	Yes (LOW RISK): The shortest prevalence period for the parameter of interest was appropriate (e.g., point prevalence, one-week prevalence, one-year prevalence), e.*g., the study was done over an appropriate time period*<br><br>No (HIGH RISK): The shortest prevalence period for the parameter of interest was not appropriate (e.g., lifetime prevalence), e.g., *there may be seasonal variation in the proportions of isolates if the sampling interval was less than 12 months.*
10	Were the numerator(s) and denominator(s) for the parameter of interest appropriate?	Yes (LOW RISK): The paper presented appropriate numerator(s) AND denominator(s) for the parameter of interest, e.g., *it is clear that numerators and denominators are correct including grouping of species and documentation of potential pathogens/contaminants consistent in both groups.*<br><br>No (HIGH RISK): The paper did present numerator(s) AND denominator(s) for the parameter of interest but one or more of these were inappropriate
	Summary risk of bias	LOW RISK OF BIAS: Further research is very unlikely to change our confidence in the estimate.<br><br>MODERATE RISK OF BIAS: Further research is likely to have an important impact on our confidence in the estimate and may change the estimate.<br><br>HIGH RISK OF BIAS: Further research is very likely to have an important impact on our confidence in the estimate and is likely to change the estimate.

Adapted from Hoy et al.(Hoy et al. 2012)

Hoy, Damian, Peter Brooks, Anthony Woolf, Fiona Blyth, Lyn March, Chris Bain, Peter Baker, Emma Smith, and Rachelle Buchbinder. 2012. “Assessing Risk of Bias in Prevalence Studies: Modification of an Existing Tool and Evidence of Interrater Agreement.” *Journal of Clinical Epidemiology* 65 (9): 934–39.

For peer review only

PRISMA-P 2015 Checklist

This checklist has been adapted for use with protocol submissions to *Systematic Reviews* from Table 3 in Moher D et al: Preferred reporting items for systematic review and meta-analysis protocols (PRISMA-P) 2015 statement. *Systematic Reviews* 2015 **4**:1

Section/topic	#	Checklist item	Information reported		Line number(s)
			Yes	No	
**ADMINISTRATIVE INFORMATION**					
**Title**					
Identification	1a	Identify the report as a protocol of a systematic review	☒	☐	Page 1
Update	1b	If the protocol is for an update of a previous systematic review, identify as such	☐	☒	
**Registration**	2	If registered, provide the name of the registry (e.g., PROSPERO) and registration number in the Abstract	☐	☒	Page 8
**Authors**					
Contact	3a	Provide name, institutional affiliation, and e-mail address of all protocol authors; provide physical mailing address of corresponding author	☒	☐	Page 1
Contributions	3b	Describe contributions of protocol authors and identify the guarantor of the review	☒	☐	Page 17
**Amendments**	4	If the protocol represents an amendment of a previously completed or published protocol, identify as such and list changes; otherwise, state plan for documenting important protocol amendments	☐	☒	
**Support**					
Sources	5a	Indicate sources of financial or other support for the review	☒	☐	Page 17
Sponsor	5b	Provide name for the review funder and/or sponsor	☒	☐	Page 17-18
Role of sponsor/funder	5c	Describe roles of funder(s), sponsor(s), and/or institution(s), if any, in developing the protocol	☐	☐	Page 17-18
**INTRODUCTION**					
**Rationale**	6	Describe the rationale for the review in the context of what is already known	☒	☐	Page 3-8
**Objectives**	7	Provide an explicit statement of the question(s) the review will address with reference to participants, interventions, comparators, and outcomes (PICO)	☒	☐	Page 8

BioMed Central
The Open Access Publisher

Section/topic	#	Checklist item	Information reported Yes	Information reported No	Line number(s)
**METHODS**					
**Eligibility criteria**	8	Specify the study characteristics (e.g., PICO, study design, setting, time frame) and report characteristics (e.g., years considered, language, publication status) to be used as criteria for eligibility for the review	☒	☐	Page 9
**Information sources**	9	Describe all intended information sources (e.g., electronic databases, contact with study authors, trial registers, or other grey literature sources) with planned dates of coverage	☒	☐	Page 10
**Search strategy**	10	Present draft of search strategy to be used for at least one electronic database, including planned limits, such that it could be repeated	☒	☐	Page 10
STUDY RECORDS					
Data management	11a	Describe the mechanism(s) that will be used to manage records and data throughout the review	☒	☐	Page 11
Selection process	11b	State the process that will be used for selecting studies (e.g., two independent reviewers) through each phase of the review (i.e., screening, eligibility, and inclusion in meta-analysis)	☒	☐	Page 11
Data collection process	11c	Describe planned method of extracting data from reports (e.g., piloting forms, done independently, in duplicate), any processes for obtaining and confirming data from investigators	☒	☐	Page 11
**Data items**	12	List and define all variables for which data will be sought (e.g., PICO items, funding sources), any pre-planned data assumptions and simplifications	☒	☐	Pages 11-12
**Outcomes and prioritization**	13	List and define all outcomes for which data will be sought, including prioritization of main and additional outcomes, with rationale	☒	☐	Pages 11-12
**Risk of bias in individual studies**	14	Describe anticipated methods for assessing risk of bias of individual studies, including whether this will be done at the outcome or study level, or both; state how this information will be used in data synthesis	☒	☐	Page 13
DATA					
**Synthesis**	15a	Describe criteria under which study data will be quantitatively synthesized	☒	☐	Page 14
	15b	If data are appropriate for quantitative synthesis, describe planned summary measures, methods of handling data, and methods of combining data from studies, including any planned exploration of consistency (e.g., I^2, Kendall's tau)	☒	☐	Pages 14-15
	15c	Describe any proposed additional analyses (e.g., sensitivity or subgroup analyses, meta-regression)	☒	☐	Page 15
	15d	If quantitative synthesis is not appropriate, describe the type of summary planned	☒	☐	Page 16

BioMed Central
The Open Access Publisher

Section/topic	#	Checklist item	Information reported		Line number(s)
			Yes	No	
**Meta-bias(es)**	16	Specify any planned assessment of meta-bias(es) (e.g., publication bias across studies, selective reporting within studies)	☒	☐	Page 16
**Confidence in cumulative evidence**	17	Describe how the strength of the body of evidence will be assessed (e.g., GRADE)	☒	☐	Page 16

For peer review only

BioMed Central
The Open Access Publisher
